# Process evaluation of an individually tailored complex intervention to improve activities and participation of older nursing home residents with joint contractures (JointConEval): a mixed-methods study

**DOI:** 10.1186/s13063-024-08652-2

**Published:** 2024-12-18

**Authors:** Regina Thalhammer, Natalie Nguyen, Gabriele Meyer, Stefanie Skudlik, Martin Müller, Katrin Beutner

**Affiliations:** 1 Faculty of Applied Health and Social Sciences, Rosenheim Technical University of Applied Sciences, Hochschulstraße 1, Rosenheim, 83024 Germany; 2 International Graduate Academy (InGrA), Institute of Health and Nursing Sciences, Medical Faculty of Martin, Luther University Halle-Wittenberg, Magdeburger Straße 8, Halle (Saale), 06112 Germany; 3 Institute of Health and Nursing Sciences, Medical Faculty of Martin, Luther University Halle-Wittenberg, University Medicine Halle, Magdeburger Straße 8, Halle (Saale), 06112 Germany; 4 Nursing Science and Interprofessional Care, Department for Primary Care and Health Services Research, Medical Faculty Heidelberg, Heidelberg University, Im Neuenheimer Feld 130.3, Heidelberg, 69120 Germany

**Keywords:** Contracture, Complex intervention, Human activities, Individually tailored intervention, International Classification of Functioning, Disability, and Health, Nursing Homes, Randomised Controlled Trials, Participation, Process evaluation

## Abstract

**Background:**

Older people with joint contractures in nursing homes often experience severe restrictions in their activities and participation. The effectiveness of an individually tailored complex intervention to improve residents’ activities and participation by incorporating the biopsychosocial perspective into nursing care using a structured facilitator approach could not be established in the JointConEval cluster-randomised controlled trial. This process evaluation aimed to systematically identify factors influencing implementation and effectiveness.

**Methods:**

The mixed-methods process evaluation analysed recruitment, implementation, mechanisms of impact, and context. Qualitative data was generated in semi-structured focus groups and in individual interviews with facilitators, nursing and social care staff, residents, relatives and guardians. Quantitative data was recorded with facilitators and 20% of nursing and social care staff using standardised documentation forms and questionnaires. Qualitative data was analysed using qualitative thematic content analysis, while the quantitative data was analysed descriptively. An interpretation was performed by combining and comparing the qualitative and quantitative results after the separate analyses.

**Results:**

The implementation was realised as planned, but the intervention did not always reach the nursing home staff, which hindered the planned change in attitude and behaviour. The attitude of the facilitators was mainly in line with the intervention. However, the intervention reached only half the residents. We identified various key influencing factors related to the context, setting and implementation agents. Nursing homes lacking facilitator support from staff or management or experiencing staff shortages and facing organisational weaknesses had difficulties in achieving the desired behavioural changes and positive primary outcomes.

**Conclusions:**

The complex intervention was delivered as planned with several factors affecting the implementation. A key influencing factor was the organisational structure and leadership of the nursing homes, which had an impact on the behaviour and motivation of the implementation agents. The findings highlight challenges in achieving behavioural changes among nursing staff in the context of long-term care in Germany. We recommend a systematic organisational context analysis for similar complex interventions in long-term care, involving stakeholders and improving leadership participation for more effective implementation.

**Trial registration:**

DRKS (German Clinical Trials Register), number DRKS00015185. Registered on 1 August 2018, https://drks.de/search/en/trial/DRKS00015185. Universal Trial Number U1111-1218–1555.

**Supplementary Information:**

The online version contains supplementary material available at 10.1186/s13063-024-08652-2.

## Background

Older people in nursing homes are frequently affected by joint contractures. The prevalence ranges between 20 and 75%, which is due to the lack of a standardised definition, different population characteristics and heterogeneous data collection methods [1].

Joint contractures are associated with reduced functional capacity, pain, limited physical mobility and bed confinement [2, 3] and may increase the dependency on care as they interfere with daily activities such as eating, dressing and walking [4]. Affected people experience limitations in highly relevant activities and their social participation [1, 4].

To improve the living situation of nursing home residents with joint contractures, we developed the PECAN (Participation Enabling Care in Nursing) intervention aimed at improving activities and participation of the target group [5–7] and evaluated its effectiveness and safety in a cluster-randomised trial [8, 9].

This paper reports the results of the process evaluation along with the respective trial to explore the implementation of PECAN. The process evaluation aimed to enhance confidence in conclusions about effectiveness and applicability by assessing delivery quantity and quality and by considering contextual factors [10]. Key components were implementation, impact mechanisms, context and their interplay, supplemented by recruitment analysis [10, 11].

### Summary of the trial

The JointConEval study (DRKS (German Clinical Trials Register), DRKS00015185) was a two-armed cluster-randomised controlled trial in nursing homes in Eastern (Halle (Saale)) and Southern Germany (Rosenheim), which was conducted from August 2018 to February 2020. The sample included 35 nursing homes (= clusters) and 562 residents. Included residents were aged 65 years or older with joint contractures affecting activities or participation. Joint contractures were defined as limited joint mobility in a major joint (shoulder, elbow, wrist, hip, knee, or ankle), diagnosed by a healthcare professional. Intervention group clusters (*n* = 18, 303 residents) received the PECAN intervention, and control group clusters (*n* = 17, 259 residents) were provided with optimised standard care. The primary endpoint assessed residents’ activities and participation at 12 months using the PaArticular Scales [12]. The secondary outcome was the health-related quality of life. The safety parameters included the number of falls by nursing home residents, fall-related consequences and physical restraints. Primary outcome analyses included 301 residents in the intervention group and 259 residents in the control group. There was no statistically significant difference in the primary endpoint after 12 months in favour of PECAN. We also found no significant differences in the secondary outcome.

#### PECAN intervention

**Fig. 1:**
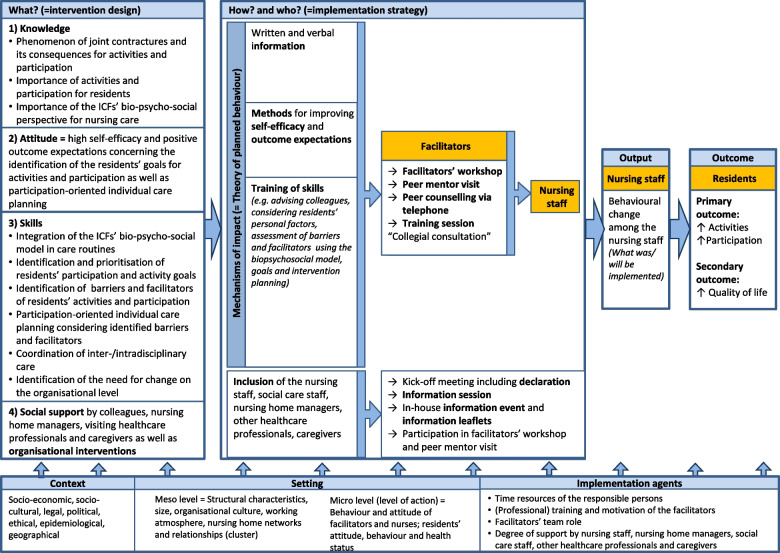
Logic model of the PECAN concept

The intervention development followed the UK Medical Research Council (MRC) framework [13]. The individually tailored intervention, based on the model of the International Classification of Functioning, Disability and Health (ICF) [14], aims to improve activities and participation for nursing home residents with joint contractures by incorporating the biopsychosocial perspective into nursing care. This involves identifying residents’ individual goals and addressing influencing factors at organisational and individual levels. See additional file 1 for intervention details and Fig. 1 for the mechanisms of action.

#### PECAN implementation strategy

**Fig. 2:**
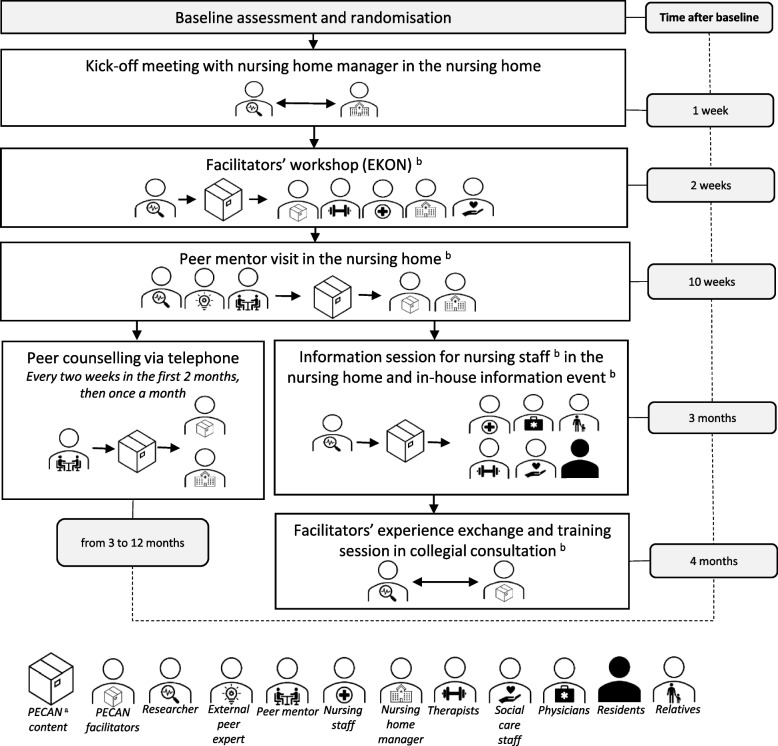
Overview of the PECAN implementation approach. ‘^a^’ indicates PECAN = Participation Enabling Care in Nursing. ‘^b^’ indicates hand-out of supporting materials

We used a structured facilitators’ approach based on the Theory of Planned Behaviour (TPB) [15] to address nurses’ and leaders’ professional attitudes and behaviour. The implementation started after randomisation (see Fig. 2). Generally, two peer mentors with a bachelor’s degree in nursing and two researchers with a master’s degree in health and nursing sciences delivered the components.

In a kick-off meeting, the researchers briefed nursing home managers about the implementation tasks and resources needed. The managers confirmed their commitment in a written declaration. Nominated nurses (at least 2 per cluster) with a minimum of three years of professional training in nursing were trained as knowledge facilitators. We also included occupational therapists and physiotherapists when no nurses were nominated. The 8-h facilitators’ workshop took place at each of the study centres and included information on joint contractures, the ICF and the implementation of PECAN. Social care staff and managers were invited to support the implementation. The peer mentoring approach comprised a 4-h on-site visit by the research team together with an external peer expert to review the residents’ care plans and to discuss organisational optimisation. Two members of the research teams offered facilitators and managers regular and on-demand telephone counselling, twice monthly for the first 2 months and then once monthly for 7 months (totalling 11 sessions per cluster). They also held consultations with managers to improve the organisational implementation (no number specified). A 45-min in-house information session about the intervention and implementation support was held for nurses and staff, and in some clusters residents and relatives were also included. This was combined with an in-house information event (2–3 h) to raise awareness about contractures, the intervention and implementation support for (visiting) staff, residents, relatives and volunteers. Four months after the initial training, a second workshop (4 h, separated from initial training due to the pilot study) focused on training facilitators on how to give advice to their colleagues during implementation. The facilitators then shared experiences and gained new impulses. We supplied printed study material at the events for the different target groups including manuals, pamphlets, posters, journal articles, brochures on health topics and logo-branded pens.

#### Optimised standard care

The nursing homes in the control group received optimised standard care, which involved a 45-min in-house presentation for staff. This session provided general information concerning the study and joint contractures. Each cluster received logo-branded pens and study pamphlets. Information on standard care in German nursing homes has been detailed in previous publications [8, 9]. We offered the intervention to the control group after the trial period and made supporting material accessible on the project website (https://bewegung-verbindet.de/materialien/).

### Aims

This study aimed to systematically evaluate the implementation of PECAN in order to understand the factors that influenced the intervention and to show how these findings relate to the results of the effectiveness evaluation.

We formulated the following research questions:

- What were the specifics of the recruitment process?
- To what extent did the clusters implement the PECAN intervention?
- What were the mechanisms of impact of the PECAN intervention?
- Which contextual factors influenced implementation and outcomes?
- How do the factors evaluated under (a) to (d) relate to the effect of the intervention?

## Methods

### Design

A convergent mixed methods design [16, 17], based on a program theory and explicated in a process evaluation concept [8], was used to explore the different research questions and gain complementary insights [11]. We followed the MRC guidance [10] focussing on the context of implementation. We also adhered to the framework of Grant et al. [11] to distinguish between processes at the cluster and individual levels. To further analyse the interactions of the intervention with contexts, we used the Context and Implementation of Complex Interventions (CICI) framework [18]. We paid special attention to the facilitators’ behaviours and attitudes aligning with the TPB [15]. Reporting followed the Template for Intervention Description and Replication (TIDieR) checklist [19], the Standards for Reporting Qualitative Research (SRQR) [20] and the guidelines of Good Reporting of a Mixed Methods Study (GRAMMS) [21].

### Sample

We recruited the participants from the nursing homes in the main trial. The process evaluation included facilitators, nursing staff (skilled nurses, nursing students, nursing assistants), social care staff (leaders and assistants), managers (directors of nursing homes and head nurses), residents, residents’ relatives, guardians and unblinded research staff.

### Data collection

Our data concept covered four domains:

- ‘Recruitment of cluster and residents’
- ‘Implementation’ with subdomains ‘Delivery to cluster’ and ‘Reach of nursing home residents’
- ‘Mechanisms of impact’ with subdomains ‘Response of cluster’ and ‘Response of residents and relatives’
- ‘Contextual factors’

Table 1 provides an overview of these domains, research questions, participants, methods and time points. Data were collected throughout the course, from recruitment to pre-, during- and post-intervention, in order to illustrate changes [10].

**Table 1:** Overview of the process evaluation measures

Domain	Research question	Participants	Methods	Stage of study
Recruitment of cluster	1. How were the nursing homes sampled and recruited?		Standardised documentation forms:	B Pre-intervention
	2. Who decided to take part in the study?	Research team	A Recruitment of nursing homes	A Recruitment
	3. Why did nursing homes participate or not?			
			Standardised questionnaire:	
		Managers (n=35)	B Cluster t0 (IG, CG)	B Pre-intervention
Recruitment of nursing home residents	4. Why did nursing home residents participate or not?		Standardised documentation forms:	
		Research team	*C* Recruitment of residents	*C* Recruitment
Implementation—delivery to cluster	5. Was the intervention delivered to the cluster as planned? (Fidelity) 6. How much of the intervention was delivered to the cluster? (Dose) 7. How was the intervention and the implementation adapted by the cluster? (Adaptation) 8. To what extent did the cluster receive the intervention? Has everyone been reached? (Reach)	Research team/ peer mentors Participants of the workshop (*n* = 45)	Standardised documentation forms: *D* Kick-off-meeting (IG) *E* Facilitators’ workshop (IG) *F* Peer mentor visit (IG) *G* Information session (IG) *H* In-house information event (IG) *I* Cluster-contact (IG) *J* Facilitators’ experience exchange (IG) Standardised questionnaire: *K* Facilitators’ workshop (IG)	*D-K* During intervention
Implementation— reach of nursing home residents	9. To what extent did the nursing home residents receive the intervention? Was everyone reached?	Peer mentors Facilitators	Standardised documentation forms: *L* Telephone peer counselling (IG) Standardised questionnaire: *M* Delivery to residents (IG)	*L* During intervention *M* Post-intervention
Mechanisms of impact—response of cluster	10. What was the attitude of the cluster regarding the intervention and the implementation strategy? 11. How was the intervention and the implementation adopted by the cluster? 12. How was the intervention integrated into the daily work routine by the cluster? 13. How was the behaviour of the cluster influenced by the intervention?	Facilitators (*n* = 45) Facilitators (*n* = 34) Facilitators (*n* = 36) Nursing and social care staff (*n* = 194) Facilitators (*n* = 33) Peer mentor (*n* = 2) Nursing and social care staff (*n* = 78) Facilitators (*n* = 30)	Standardised questionnaire: *K* Facilitators’ workshop (IG) *P* Facilitators’ exchange (IG) *Q* Facilitator t1 (IG) *O* Nursing and social care staff t2 (IG) *R* Facilitator t2 (IG) *S* Peer mentor (IG) Semi-structured focus group interviews/individual interviews: *T* Nursing and social care staff (IG) *U* Facilitators (IG)	*K, P, Q* During intervention *O, R, S, T, U* Post-intervention
Mechanisms of impact—response of nursing home residents and relatives	14. How were nursing home residents affected by the intervention? 15. How did nursing home residents react to the intervention?	Nursing and social care staff (*n* = 78) Facilitators (*n* = 30) Relatives/guardians (*n* = 11) Residents (*n* = 35) Peer mentors	Semi-structured focus group interviews/individual interviews: *T* Nursing and social care staff (IG) *U* Facilitators (IG) Semi-structured individual interview: *V* Relatives/guardians (IG) *W* Residents (IG) Standardised documentation forms: *L* Telephone peer counselling (IG)	*T, U, V, W* Post-intervention *L* During intervention
Context	16. What is the context in which the trial is conducted? 17. What are the barriers and facilitators of implementation in each context?	Managers (*n* = 35) Managers (*n* = 35) Nursing and social care staff (*n* = 394) Facilitators (*n* = 33) Nursing and social care staff (*n* = 78) Facilitators (*n* = 30) Relatives/guardians (*n* = 11) Residents (*n* = 35)	Standardised questionnaire + D-OCAI: *B* Cluster t0 (IG, CG) *X* Cluster t2 (IG, CG) *O* Nursing and social care staff t2 (IG, CG) *R* Facilitator t2 (IG) Semi-structured focus group interviews/individual interviews: *T* Nursing and social care staff (IG) *U* Facilitators (IG) Semi-structured individual interview: *V* Relatives (IG) *W* Residents (IG)	*B* Pre-intervention *X, O, R, T, U, V, W* Post-intervention

*Abbreviations*: *IG* Intervention group, *CG* Control group *t0* Baseline, *t1* 6 months after baseline, *t2* 12 months after baseline, *D-OCAI* German version of the Organizational Culture Assessment Instrument [22]

The qualitative data collection, which took place in the intervention clusters, comprised focus groups with the facilitators in the study centres and individual telephone interviews for those unable to attend the focus groups. We interviewed nursing and social care staff in focus groups on-site or by telephone if the participants could not be reached otherwise. In addition, residents were interviewed on-site and relatives or guardians by telephone. The semi-structured interview guides covered implementation experiences, intervention planning, target group involvement and reactions and an evaluation of PECAN. Interviews were conducted by experienced researchers involved in the implementation, thus fostering trust with facilitators and managers. Quantitative data collection took place at baseline, 6 months, and 12 months, using paper-based questionnaires for the facilitators and a random sample of 20% of nursing and social care staff. The researchers documented the implementation components using standardised paper documentation. We assessed the organisational culture of the intervention and the control group clusters at baseline and after 12 months using the German version of the Organizational Culture Assessment Instrument (D-OCAI) [22]. The D-OCAI, based on the Competing Values Model [23], includes four domains (dominant characteristics, management of employees, organisation glue and criteria of success), each with four items rated on a 5-point scale. The results are mapped in four quadrants for the organisational culture types: dynamic, entrepreneurial adhocracy (Open Systems), people-oriented clan culture (Human Relations), process-oriented hierarchy (Internal Processes) and results-oriented market culture (Rational Goals).

### Data analysis

Following the convergent design, we analysed each data set separately before merging and comparing the results to interpret how the data align, differ or combine to enhance understanding [16]. We conducted a side-by-side analysis, first evaluating qualitative findings and then quantitative results. All interviews and focus groups were audio-recorded, transcribed verbatim [24] and then pseudonymised and anonymised post-analysis. Transcripts underwent qualitative thematic content analysis [25] using MAXQDA 2022. Firstly, two researchers (RT, NN) independently applied categories derived from research questions and interview guides, refining them inductively with subcodes. This independent process aimed to enhance confirmability. Subsequently, categories were discussed and refined for clarity, comprehensiveness and coherence with a third researcher (KB) to ensure dependability. Quotes were translated by a native speaker to ensure the trustworthiness of the results [26]. Quantitative data were analysed descriptively (frequencies, percentages, means, range) using IBM SPSS Statistics version 27, addressing the different domains outlined in Table 1.

### Ethical considerations

The Ethics Committees of the Martin Luther University Halle-Wittenberg (Reference No. 2018–63, June 2018) and the Ludwig-Maximilians-University Munich (Reference No. 18–356, July 2018) approved the process evaluation as a part of the c-RCT. Participants in the written surveys gave their consent by returning the questionnaires. We obtained written informed consent from participants in the individual interviews and focus groups.

## Results

### Sample

The qualitative data comprised four focus groups with facilitators (58–85 min, mean: 71 min), seven telephone interviews (7–35 min, mean: 23 min), 15 focus groups with nursing and social care staff (14–65 min, mean: 42 min) and 4 telephone interviews (10–22 min, mean: 15 min). Individual interviews were conducted with 35 residents (5–34 min, mean duration: 16 min) and 10 relatives or guardians (7–22 min, mean duration: 13 min). An overview of the sample characteristics is presented in additional file 2. Quantitative data were collected from facilitators, nursing and social care staff and management; see Table 1 for participant numbers. Additional file 3 provides an overview of the quantitative sample characteristics for nursing and social care staff.

### Recruitment procedure

We contacted 255 nursing homes (Halle (Saale): *n* = 165, Rosenheim: *n* = 90) through e-mail invitations and subsequent telephone calls between February and December 2018, recruiting 35 clusters. In most cases (*n* = 229, 89.8%), the decision regarding participation was made by the nursing home directors, the head nurses or both. Reasons for declining participation included resource limitations (29.5%), involvement in other projects (17.6%) or lack of interest (14.8%). Participating clusters stated interest in the topic, hoped to improve care quality or to gain knowledge. We invited 731 residents identified by the head nurses, of which 578 consented, and 562 were enrolled. Sample size calculation required 18 or 19 residents to be included per cluster, but due to low recruitment, we enrolled clusters with fewer residents, as predefined in the study protocol. The main reasons for non-participation (*n* = 169) were guardian refusal (32.5%), failure to meet inclusion criteria (30.2%) and resident refusal (24.9%).

### Scoring of the delivery of the intervention to cluster

**Fig. 3:**
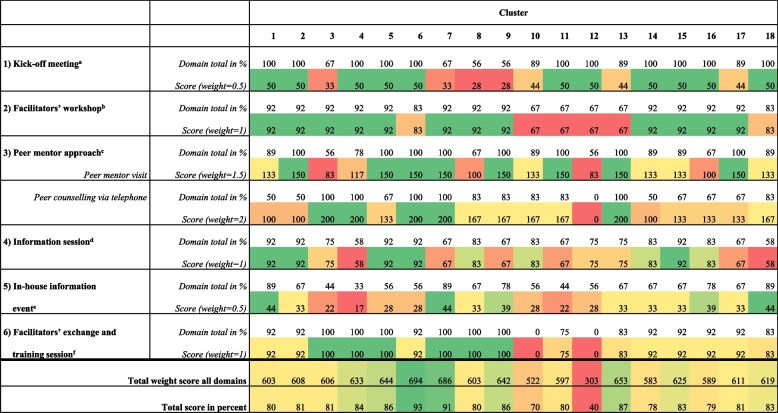
Short overview of the delivery in the clusters. Colour gradient from green, yellow to red: good delivery = green; fair delivery = yellow; poor delivery = red. ‘^a^’ indicates rating: participation of at least one manager; the number of nominated facilitators; declaration was signed by leaders; meeting conducted according to protocol; satisfaction with the delivery. ‘^b^’ indicates rating: number of trained facilitators in relation to total number of facilitators nominated; achievement of learning objectives; agenda and content according to protocol; satisfaction with delivery. ‘^c^’ indicates rating of peer mentor visit: adequate preparation of the cluster at the individual level and at the organisational level; agenda and content according to protocol; satisfaction with delivery; rating of peer counselling via telephone: number of telephone consultations with facilitators and with managers; number of facilitators counselled in relation to total number of facilitators. ‘^d^’ indicates rating: invited groups and type of invitation; achievement of learning objectives; session conducted according to the protocol; satisfaction with delivery. ‘^e^’ indicates rating: invited groups and type of invitation; information event conducted according to the protocol; satisfaction with delivery. ‘^f^’ indicates rating: achievement of learning objectives; agenda and content according to protocol; satisfaction with delivery

We analysed implementation data for 18 intervention clusters, focusing on ‘fidelity’, ‘dose’, ‘adaptation’ and ‘reach’, using descriptive statistics. Figure 3 provides an overview of the delivery and implementation level in each cluster, detailed further in additional file 4. To summarise the level of implementation, we developed a scoring system (additional file 5). The domains were scored and weighted based on their relevance to the intervention program, with consensus from all involved researchers: The kick-off meeting and the in-house information event each have a maximum score of 50. The facilitators’ workshop, information session and facilitators’ exchange and training session each have a maximum of 100 points. The peer mentor visit has a maximum of 150 and peer counselling via telephone has a maximum of 200. After each implementation component, delivering researchers at the respective study centre completed the scoring system as a collective assessment on paper-based standardised forms.

### Delivery and quantitative evaluation of cluster response

#### Kick-off meeting

The kick-off meeting was conducted in all clusters following the protocol to 83.3% (*n* = 15). One meeting ended early, and two were conducted by telephone.

#### Facilitators’ workshop

Five 8-h workshops were held at the two study centres (Halle: 3, Rosenheim: 2). Out of the 56 registered participants, 45 (80%) attended (per cluster: median 2; min 1; max 5). Characteristics of workshop participants are provided in additional file 6. Reasons for not attending were illness (*n* = 4) or time limitations (*n* = 5). One person withdrew and another did not provide a reason for non-attendance. Attendees (*n* = 43) rated the event on average as ‘good’ (SD 1.18; min. 1, max. 5) on a scale from 1 to 6 (1 = very good, 2 = good, 3 = satisfactory, 4 = sufficient, 5 = insufficient, 6 = poor). Expectations were fully met for *n* = 33 (75%), partially for *n* = 4 (9.1%) and not at all for *n* = 7 (15.9%). Some participants noted a lack of innovative content and relevance to their practice. While 36 participants (80%) felt mostly confident in implementing tasks, 5 (11.1%) were very confident, and 4 (8.9%) were mostly not confident.

#### Peer mentor approach

The peer mentor visit was conducted in all clusters. In three clusters, researchers noted challenges with implementing objectives and observed a negative attitude towards the intervention. Conversely, a motivated attitude towards PECAN was observed in the other clusters. Facilitators (*n* = 33) rated the visit as ‘good’ (SD 0.75; min. 1, max. 4) on a scale from 1 to 6 (1 = very good, 2 = good, 3 = satisfactory, 4 = sufficient, 5 = insufficient, 6 = poor). Six months after baseline, 69.7% (*n* = 23) of the facilitators reported that the peer mentor visit was a helpful support in applying the workshop content.

Of the 11 planned telephone counselling sessions per cluster, between 0 and 18 were realised (median: 10 sessions, in total 181 of 198 sessions = 91%) lasting 92 h overall (per cluster: median: 5.8; min. 0; max. 8.47 h). Only one cluster had no consultations, as they were deemed unnecessary. Facilitators rated telephone counselling as ‘very good’ (SD 0.4; min. 1, max. 2) on a scale from 1 to 6 (1 = very good, 2 = good, 3 = satisfactory, 4 = sufficient, 5 = insufficient, 6 = poor) 12 months after baseline with 88.9% (*n* = 24) finding it helpful for addressing practical issues. Six clusters requested additional on-site counselling sessions lasting 45 to 155 min.

#### Information session and in-house information event

One information session and an in-house information event were held per cluster as planned, but attendance was limited for unknown reasons.

#### Facilitators’ experience exchange and training session

Four sessions were conducted as scheduled (Halle: 2, Rosenheim: 2) with 30 (77%) of 39 facilitators. Reasons for absence included staff-related issues (*n* = 3), illness (*n* = 2), traffic delays (*n* = 2) and lack of interest (*n* = 2). All 34 participants were very or mostly satisfied with the content with 31 rating it ‘very good’ (SD 0.45; min. 1, max. 2) on a scale from 1 to 6 (1 = very good, 2 = good, 3 = satisfactory, 4 = sufficient, 5 = insufficient, 6 = poor).

#### Supportive material (not displayed in Fig. 3)

The delivery of information and training material was mainly standardised. Each cluster received 25 logo-branded pens, study pamphlets and PECAN flyers for residents, relatives, staff, therapists and physicians (120 pamphlets per cluster for clusters 1 to 9 and 170 pamphlets in clusters 10 to 18). We supplied the participants with handouts at each event. The clusters were given a motivational poster per ward for residents and relatives and a PECAN poster for healthcare professionals. The facilitators received two pocket cards per resident to document their individual goals, along with motivational cards for residents (mean per cluster: 8.5), and journal articles (mean per cluster: 14.6).

### Qualitative evaluation of cluster response and attitude

The facilitators generally responded positively to PECAN and felt well-prepared, with their attitudes influencing its adoption. Increased knowledge gave them more confidence for the implementation.

‘So with the perspective and also with the background knowledge, I have found that I myself am simply more courageous in the implementation. Also that you have more (…) confidence, even with those residents […], where you might have hesitated before.’ (Facilitator focus group, Cluster 5).

Peer mentors confirmed varying attitudes towards PECAN among the facilitators. On a scale from 1 (‘does not correspond to PECAN at all’) to 6 (‘corresponds fully’), the facilitators had a mean rating of 4.53 (SD: 1.54), indicating a range between ‘corresponds rather to PECAN’ and ‘corresponds mostly to PECAN’. Even those less aligned with PECAN principles showed high implementation rates in some clusters (Fig. 3).

Twelve months after the baseline, 46.9% of the nursing and social care staff reported feeling well-informed about PECAN, while 56.7% found the information session to be understandable. Half of them stated that the facilitators were consistently available for advice. Staff attitudes towards PECAN varied across clusters and did not always align with those of the facilitators.

‘We have also had employees who have said directly to my face “It is much quicker for me if I do that. If I take that off their hands [preparing breakfast, pouring drinks], I can tick it off much more quickly.” […] And then you just stand there stunned, because you actually talked about it beforehand […], but then it is simply ignored.’ (Facilitator focus group, Cluster 9).

Different approaches were used to integrate PECAN into practice, such as including it in case conferences and collaborating with physiotherapists to ensure continuity. Some clusters applied PECAN to residents without joint contractures, emphasising a broad eligibility. The facilitators noted that integrating PECAN into routines did not pose a time problem, as processes become automatic after an initial restructuring period.

‘So we accepted all the residents because we just think that everyone is entitled to it and because I think it is more difficult for the staff to concentrate on one resident in particular […]. That is why we didn’t have a time problem […] it’s been assimilated and become an automatic process.’ (Facilitator focus group, Cluster 1).

The facilitators believed that the nursing staff, having been sensitised to the topic, would proactively implement PECAN in the future, perceiving the benefits as outweighing the negatives. Most clusters intend to continue PECAN after the study, indicating a change in professional behaviour.

### Delivery and response of nursing home residents and relatives

**Fig. 4:**
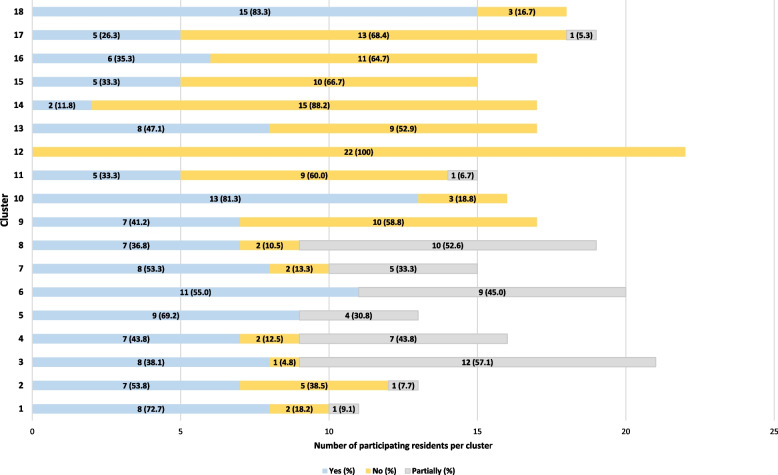
Delivery to residents per cluster

The facilitators delivered PECAN as planned to 43.5% of the residents (*n* = 131), partially to 16.9% (*n* = 51) and not at all to 39.5% (*n* = 119) of the intervention group. Figure 4 illustrates the delivery of PECAN to residents per cluster, assessing individualised care planning. In eight clusters, PECAN was delivered to less than half of the residents.

Clusters introduced organisational changes to improve the residents’ activities and participation, including redesigning spaces and creating attractive seating areas. Staff focus groups highlighted individual-level changes in line with the ICF model, with 525 interventions fully implemented, though some only occurred once. Table 2 provides the number of individual interventions and quotes illustrating changes in activities and participation.

**Table 2:** Changes in activities and participation at the individual level

ICF dimension	Number of planned interventions (%) *n* = 768	Number of fully implemented interventions (row percentage) *n* = 525	Quotes related to changes in residents regarding interventions at the individual level
Body functions	14 (1.8)	13 (92.9)	*‘And she has become so mentally fit too, yes, because that also helps. And with Ms. (name of resident) you noticed that she really has become increasingly mobile and mentally fitter.’ (Nursing and social care staff focus group, Cluster 13)*
Activities and participation	296 (38.6)	210 (71.0)	*‘And they beam at you when you come and praise them. You walk across the living area and someone comes up to you and you say “Oh man, that wouldn’t have been possible six months ago” and then you make small talk and praise him (…).’ (Facilitator focus group, Cluster 5)* *‘They have now formed a kind of walking group. Three of them go for a walk in the afternoon.’ (Facilitator individual interview, Cluster 14)*
Environmental factors			
Products and technology	51 (6.6)	25 (49.0)	*‘We receive medical aids *via* our cooperation partner and have now also been able to bring a second cooperation partner on board *via* our wound care, where we also get items on loan and are supplied with aids much more quickly.’ (Facilitator focus group, Cluster 1)*
Support and relationships	150 (19.5)	97 (64.6)	*‘I just know that the relatives were really happy about the resident, because they also got better access to him again. I mean, this is a closed ward, they (the residents) all live in their own world and they (relatives) now have a completely different access to him: what can they do and how can they approach him. And they were also quite happy and grateful about this (…).’ (Facilitator focus group, Cluster 18)*
Services, systems and policies	189 (24.6)	129 (68.2)	*‘And then it was also about what you can do that is good for them. For example, one of my residents wanted to have massages and physiotherapy, and Mrs. (name of the facilitator) brought this up with me, because I have to clarify this with the physician. Then a prescription was issued for her to get massages, which did her a world of good.’* (*Nursing and social care staff focus group, Cluster 11*)
Personal factors	68 (8.9)	51 (75.0)	*‘She (the resident) was also very reserved towards us, she isn’t like that anymore, she is very open to us now, and she is trustful (…).’* (*Facilitator individual interview, Cluster 14)* *‘(…) he (the resident) is more motivated now and generally participates more. He no longer just sits there, sleeps, and says, “No thanks,” only reading the newspaper aloud, but no, he participates, he likes to join in. He is also enthusiastic about things that happen downstairs or when something is going on outside (…).’* (*Facilitator individual interview, Cluster 14)*
Discrepant responses: generally no improvement	No data available	No data available	*‘And it turned out, and I think this will also come out in the results, that it didn’t help that much. Because the residents have become older in the meantime.’* (*Nursing and social care staff focus group, Cluster 3*) *‘I only know that with our two residents these interventions were carried out and with one person we found out that this cannot be improved.’* (*Nursing and social care staff focus group, Cluster 11*)

Staff reported more successes in improving activities and participation than relatives, guardians and residents, who perceived fewer changes. However, some relatives noticed positive changes in residents’ living situations, which improved relationships.

‘What I’ve noticed is that he’s been much more motivated and fit lately. I mean, my father was always very quiet and never talked much. […] I have to say that it hasn’t been like that lately. He suddenly started talking a lot. He told me what he was doing and so on […]. Because of this, we had a very good relationship with each other again recently.’ (Relative individual interview, Cluster 3).

Residents often attributed outcome assessment interviews as part of the study rather than recognising changes in daily life and care due to PECAN.

‘No, it’s all stayed the same. I also have osteoarthritis. But it’s no worse. I am satisfied.’ (Resident individual interview, Cluster 10).

This challenge extended to some relatives and guardians not directly involved in planning interventions, despite recommendations to facilitators. According to staff, residents viewed it as a new social care concept with smaller and more individual groups. Some residents did not explicitly notice any changes attributed to the study, expressing that nothing substantial had occurred. However, one relative confirmed positive results, such as improved mobility, which the resident did not associate with the study.

### Context

#### Changes in context

**Fig. 5:**
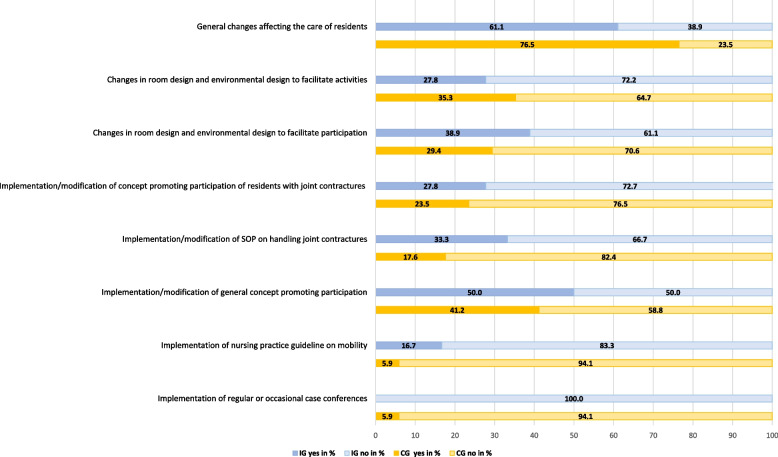
Changes of context in the intervention group and control group. Abbreviations: IG, intervention group; CG, control group; SOP, standard operating procedure

Nursing home characteristics at baseline are detailed in additional file 7. The intervention and control groups showed similar baseline criteria, ensuring comparability. Over the 12-month period, the intervention group showed more frequent implementation of non-PECAN-related changes than the control group, as depicted in Fig. 5.

There were minimal differences in organisational culture between the two groups. Exemplary cluster analysis results from nursing home managers indicated that most clusters aligned with a collaborative clan culture (Human Relations); see additional file 8.

#### Barriers and enabling factors

We assessed barriers and enabling factors experienced by staff during implementation through questionnaires on perceived resources and social support at the 12-month follow-up (see additional file 9). Responses by nursing and social care staff indicated that the intervention group felt more supported by healthcare professionals than the control group. The facilitators also reported positive feedback on interprofessional cooperation during the focus groups.

‘We were actually all in the same boat from the beginning, whether it was the management, the therapists, or colleagues, they all pulled together.’ (Facilitator focus group, Cluster 5).

However, they also noted challenges in interprofessional cooperation and irregular implementation of measures. They identified communication issues as barriers to maintaining consistent collaboration with the social care and nursing staff.

‘So on the whole I really have to say that I found the cooperation with the nursing staff to be rather negative. Things were implemented or attempted to be implemented and that worked 5 or 6 times and […] due to changes of duty, communication was lost at some point, […] and then the topic was shelved again.’ (Facilitator focus group, Cluster 8).

**Fig. 6:**
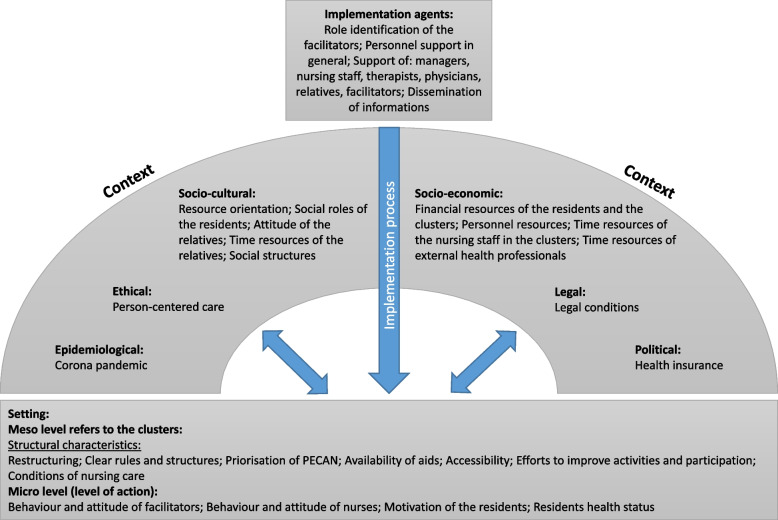
Overview of the context analysis adapted from Pfadenhauer et al. [18]

The qualitative analysis highlighted the interplay of context, setting and implementation agents that influenced the implementation as shown in Fig. 6.

Staff discussed the impact of socio-economic factors such as financial resources of residents and clusters and staff availability. While some interventions required extra funding, others did not.

‘Well, we have managed it with some residents, even with one resident, who made a little trip […]. But of course, that’s another thing, he needed his own financial means.’ (Facilitator individual interview, Cluster 15).

In the socio-cultural context, staff, relatives and residents emphasised the influence of social roles. Challenges arose when residents held a deficit-oriented view of old age requiring additional efforts from staff or relatives.

‘A change, yes, but at eighty-eight you don’t need much exercise. You’re glad to have your peace.’ (Resident individual interview, Cluster 11).

This was linked to highly supportive or restrictive social structures in old age. Some residents felt lonely, while others formed new friends in the nursing home.

‘I have a friend. She’s there too. She’s the same age as me and then we just talk a bit about [our employment] now and then (…).’ (Relative individual interview, Cluster 11).

Legal and political conditions created implementation obstacles.

‘Of course, it is also a question of the health insurance companies. So even if you have good ideas and you request approval (…), it is partly not approved by the health insurance company.’ (Nursing and social care staff focus group, Cluster 6).

The ‘implementation agents’ domain involves information dissemination and facilitators’ support from staff and relatives. Facilitators reported that while effective communication enhanced performance, insufficient management support and limited staff involvement hindered successful implementation.

‘Because it was rather forced on me and I would have liked more support from the management level. […] Well, when I caught the staff, they were actually always ready to listen. But it was really difficult to get hold of them first and then get half an hour of their time. As I said, I was pretty much left alone.’ (Facilitator focus group, Cluster 2).

The meso-level setting included factors like medical aid availability and the impact of resources on care conditions. Nursing staff emphasised the importance of supportive relatives and a stable care setting.

‘[…] if you have a good general practitioner who says “OK, I’ll write down physiotherapy so that we can promote it additionally”, who then maybe says “OK, we’ll write down other aids” [then it works]. You also have physicians who say “Such nonsense, we don’t need it!”’ (Nursing staff focus group, Cluster 4).

The micro-level setting encompasses the attitudes of facilitators, nursing staff and residents’ motivation and health. Facilitators noted that some staff held a negative view of ageing, which hindered implementation.

‘The nursing staff and the assistants still have this mantra in their heads: “They are all old, they all need help and we do as much as we can”.’ (Facilitator focus group, Cluster 2).

The residents’ deteriorating health posed challenges for planning and execution. The success of a complex intervention in nursing homes depends on various factors that can either support or obstruct implementation.

### Result interpretation

The effectiveness evaluation indicated that most intervention clusters had a non-significant trend towards improvement of the primary outcome [9]. Table 3 compares this outcome with process evaluation data.

**Table 3:** Comparison of primary outcome with process evaluation data

	**1**	**2**	**3**	**4**	**5**	**6**	**7**	**8**	**9**	**10**	**11**	**12**	**13**	**14**	**15**	**16**	**17**	**18**	**Overall change intervention group (*n *= 301)**	**Overall change control group (*n *= 259)**
**Primary outcome** ^**a**^																				
**Participation scale**																				
Baseline: mean	39	46	42	42	35	33	46	35	33	53	51	52	52	32	40	40	41	47		
12 months after baseline: mean	19	39	32	30	18	30	39	37	26	39	47	48	39	32	38	38	39	59		
*Absolute change of Participation Rasch score from baseline*																				
Mean	−22	−3	−11	−10	−19	−3	−7	5	−7	−13	−2	−2	−7	0	−1	−1	2	14	−4	−3
**Activities scale**																				
Baseline: mean	52	58	58	60	53	47	54	54	47	60	55	59	59	32	47	55	54	62		
12 months after baseline: mean	43	51	54	47	41	44	50	51	42	55	56	55	56	42	48	57	56	62		
*Absolute change of Activity Rasch score from baseline*																				
Mean	−12	−5	−4	−12	−11	−3	−7	−2	−6	−1	1	−1	0	10	3	2	3	4	−1	0
**Delivery of the intervention to cluster**																				
Delivery score (for details, see Fig. 3)	88	87	77	80	88	88	87	80	83	64	75	52	81	84	87	84	83	83		
**Delivery to residents**																				
Number of participating residents per cluster (*n* = 301)	11	13	21	16	13	20	15	19	17	16	15	22	17	17	15	17	19	18		
Delivery to residents fully achieved	8	7	8	7	9	11	8	7	7	13	5	0	8	2	5	6	5	15		
In %	72.7	53.8	38.1	43.8	69.2	55.0	53.3	36.8	41.2	81.3	33.3	0	47.1	11.8	33.3	35.3	26.3	83.3		
*Organisational level*																				
Number of planned interventions at organisational level (*n* = 153)	11	8	5	11	9	5	8	3	9	2	14	7	3	4	19	10	12	13		
Number of successfully implemented interventions at organisational level	9	5	1	10	8	3	0	2	4	2	3	3	0	0	12	5	9	4		
in %	81.8	62.5	20	90.9	88.9	60	0	66.7	44.4	100	21.4	42.9	0	0	63.2	50	75	30.8		
*Individual level*																				
Number of planned interventions at individual level (*n* = 768)	46	32	38	26	27	40	30	34	33	69	59	0	63	28	47	49	42	105		
Number of successfully implemented interventions at individual level (*n* = 525)	42	11	26	16	27	34	23	31	22	44	35	0	31	11	22	37	24	89		
in %	91.3	34.4	68.4	61.5	100	85.0	76.7	91.2	66.7	63.8	59.3	0	49.2	39.3	46.8	75.5	57.1	84.8		
Mean number of individual-level interventions per resident (without organisational level)	5.3	1.6	3.3	2.3	3.0	3.1	2.9	4.4	3.1	3.4	7.0	0.0	3.9	5.5	4.4	6.2	4.8	5.9		

^a^Score ranges between 0 (no problem) and 100 (complete problem)

The analysis shows trends towards improved activities and participation in the clusters where at least two facilitators received telephone counselling. However, the number of sessions rated as very positive by the facilitators did not appear to impact the outcome. The primary outcome seemed to improve in six clusters that received additional on-site counselling sessions. Surprisingly, clusters that did not participate in the facilitators’ experience exchange and training sessions also showed this trend. A higher delivery score, along with a high delivery rate among residents and the implementation rate of interventions, appear to positively influence the primary outcome. Interestingly, the average number of interventions per resident does not seem to correlate with a better primary outcome; even clusters with fewer interventions showed improvement trends.

In clusters where facilitators lacked support from staff or management, changes in behaviour and primary outcome could not be achieved. The facilitators’ roles in these cases did not align with PECAN standards. Decreased activities and participation were evident amid staff shortages, absences and turnover. Weaknesses in output and outcomes occurred when management was absent or changed during the trial and if facilitators did not participate voluntarily. Organisational influences, such as communication problems, team conflicts and structural issues, hindered desired behaviour and outcomes. Conversely, clusters with stable organisational structures showed positive effects.

## Discussion

Our study assessed the implementation PECAN intervention aimed at improving activities and participation in nursing home residents with joint contractures, focusing on mechanisms of impact and contextual factors. The main goal was to gain insight into why the intervention was not able to alter the primary outcome in the intervention group. The dimensions of the intervention implementation—fidelity, dose, adaptation and reach—were mainly realised as planned. The nurses facilitating participation-orientated care successfully integrated PECAN into their daily routines. However, nursing staff showed minimal behavioural and attitudinal change, while organisational structures and leadership appeared to strongly influence implementation and outcome.

Achieving a behavioural change among nursing staff in German long-term care proved challenging. Although over half of the surveyed nursing and social care staff felt well informed about PECAN, the low attendance at information sessions raised concerns about the effective outreach. High turnover and absences hindered the creation of a common understanding of activities and participation which is crucial for successful implementation [27, 28].

Several facilitators mentioned feeling unsupported by nursing staff during implementation, describing themselves as ‘lone wolves’ despite assurances from management. Peer mentors noted that some facilitators lacked the skills to engage staff effectively, despite the managers having received a qualification profile in advance. Facilitators may have been overburdened. Peer counselling, a key component of the intervention, lacked evaluation, leaving gaps in understanding its implementation. Even without this extra task, nursing staff often face high workloads, which could be alleviated by social support and adequate resources [29]. Studies cited lack of expertise and motivation as barriers to implementation, emphasising the need to increase staff motivation [29, 30]. Facilitators noted that staff took over residents’ activities to save time, undermining participation and autonomy, a trend confirmed by research showing unnecessary involvement in daily tasks [31].

In focus groups and interviews, organisational structures were often cited as major obstacles to implementation. Responsibility was divided between nursing staff (medical measures, support in daily activities) and social care staff (support with activities and participation), with little regular communication. This challenge is also reported in the literature [32]. Better collaboration occurred when management supported joint efforts, such as including social care staff facilitator workshops or involving the head of social care. Successful cultural change relies on involvement from all staff levels, with managers emphasising the significance of widespread engagement through training and continuous communication [33]. While we focused on nursing staff, a broader reach could be achieved by involving other professions and forming a working group with active participation from management at all levels.

Van der Zijpp et al. [34] recommended establishing supportive structures to strengthen the management facilitator interaction during implementation, providing skills development and resilience building for facilitators. For PECAN, role profiles for both facilitators and managers could be created, alongside workshops to assess and develop skills. Stakeholder analysis and working groups might also help establish supportive structures [35].

Studies indicate that staff-related factors like turnover, absenteeism, workload, and managerial support are both major barriers and facilitators in implementing nursing home interventions [36, 37]. Organisational factors, such as funding, logistics and infrastructure also matter. High staff turnover, particularly among managers, disrupts continuity [38]. Clusters with better interpersonal dynamics, motivation and managerial support showed more success through an interprofessional learning culture and readiness for change [36, 39].

Tailoring the intervention to residents` goals was supported by intensive peer mentor guidance for facilitators. However, less than half of the residents received PECAN-defined interventions which varied widely, from one-off activities like attending a football match to regular ones like gardening. The minimal changes observed by residents were surprising but may be due to the difficulty in standardising tailored interventions and defining a minimum effective dose. Additionally, the outcome measures may not have fully captured the residents’ current needs. Environmental factors, such as culture, social dynamics and residents’ health and attitude, heavily influence activity choices [40].

Although positive changes were reported by facilitators, staff and relatives, these were not reflected in the primary or secondary outcomes, highlighting the need for further research into more suitable tools for measuring participation and satisfaction with participation [41].

This process evaluation highlights the critical role of organisational structures and leadership in successful implementation. Effective implementation requires a solid understanding of the organisation, close collaboration between management and staff, and organisational readiness for change, ensuring that responsibility for success is shared and embedded at the organisational level [30, 36].

### Methodical strengths and limitations

This study demonstrates several strengths. A comprehensive and structured approach was used, aligning with key recommendations for evaluating complex interventions. Detailed documentation of implementation components provided insights into challenges, success factors and areas for improvement [11]. As recommended [10], the inclusion of diverse target groups, such as residents and relatives, offered valuable perspectives on their experiences with PECAN.

However, there were limitations. Interviews with managers were only conducted if they served as facilitators, potentially missing organisational insights [18]. Since no qualitative interviews were conducted in the control group, we relied solely on the final questionnaire to assess changes during the intervention period and identify factors influencing activities and participation. The chosen theory may not have been optimally applied, as key dimensions of the TPB were not reflected in all implementation agents. Greater emphasis on behavioural control, social norms and organisational attitudes was necessary. Additionally, TPB does not address the gap between intention and behaviour, suggesting that more complex theories might be more appropriate. Interventions to change professional behaviour are complex and embedded in intricate organisational and policy contexts, emphasising collective action over individual processes. Successful interventions in such settings often induce normative and relational restructuring and validate new practice norms through experience [42].

The D-OCAI [22] was unable to monitor changes in organisational culture in our study with static results contradicting qualitative findings on the impact of leadership and organisational structure. Addressing organisational culture necessitates a focused mixed-methods approach, such as concept mapping and pattern matching, in order to explore core dimensions like leadership, communication systems and openness [43] which should be considered in future studies.

## Conclusions

This process evaluation identifies factors that influenced the implementation of the PECAN intervention. Challenges in achieving behavioural and attitudinal changes among nursing home staff were linked to organisational structures and leadership, significantly impacting overall implementation success. Management support and behaviour emerged as key factors. Although the intervention components were implemented in a standardised but also individualised manner, their implementation varied significantly. Positive changes reported by staff and relatives did not align with the primary and secondary outcomes, suggesting a need for further research on the validity of outcome assessments related to participation and satisfaction.

To support the autonomy of nursing home residents, it is vital to establish a common understanding of participation in the long term. We recommend conducting a systematic analysis of the organisational context for similar interventions to better engage key stakeholders and improve structured participation from leaders.

## Supplementary Information

Additional file 1. Strategies of the PECAN intervention.

Additional file 2. Sample characteristics of interview and focus group participants.

Additional file 3. Sample characteristics of questionnaire participants: Nursing and social care staff.

Additional file 4. Detailed overview of the delivery in the clusters.

Additional file 5. Delivery scoring system.

Additional file 6. Characteristics of facilitators’ workshop participants.

Additional file 7. Baseline characteristics of nursing homes.

Additional file 8. Organisational culture of the clusters of the intervention group (IG) and control group (CG) at baseline (t0) and after 12 months (t2).

Additional file 9. Perceived resources and social support in the clusters from the perspective of nursing & social care staff and facilitators to promote activities and participation after 12 months.

## Data Availability

The datasets generated and analysed during the current study are available from the corresponding author upon reasonable request. Materials used for the implementation are available on the project homepage https://bewegung-verbindet.de/materialien/.

## Abbreviations

CICI: Context and Implementation of Complex Interventions Framework
c-RCT: Cluster-randomised controlled trial
CG: Control group
DRKS: German Clinicals Trials Register
GRAMMS: Good Reporting of a Mixed Methods Study
ICF: International Classification of Functioning, Disability and Health
IG: Intervention group
MRC: UK Medical Research Council
OCAI: Organizational Culture Assessment Instrument
PECAN: Participation Enabling Care in Nursing
SD: Standard deviation
SOP: Standard operating procedure
SRQR: Standards for Reporting Qualitative Research
TIDieR: Template for Intervention Description and Replication
TPB: Theory of Planned Behaviour
