# Supplementary material for: Process evaluation of an individually tailored complex intervention to improve activities and participation of older nursing home residents with joint contractures (JointConEval): a mixed-methods study
Source: Trials. 2024 Dec 18;25:831. doi: 10.1186/s13063-024-08652-2 (PMC11654093; doi:10.1186/s13063-024-08652-2)
Supplement: Supplementary file 1 — Additional file 1. Strategies of the PECAN intervention. [file 13063_2024_8652_MOESM1_ESM.docx]

Additional file 1. Strategies of the PECAN intervention

| Actions to improve activities and participation in individuals with joint contractures:   - Integrate the biopsychosocial perspective of the ICF into daily care activities - Identify and prioritise tailored goals for the residents’ activities and participation - Identify influencing factors to the residents’ activities and participation - Adapt care planning with a focus on activities and participation considering the identified influencing factors | Improvement of activities and participation can be achieved by targeting ICF components:   - Improve impaired body functions and structures - Resource-oriented promotion of activities to improve the residents’ independence in daily life and reduce limitations - Consider the residents’ personal factors - Address hindering environmental factors |
| --- | --- |
| *Detailed approach to achieve the intervention goals* | |
| Individual level   - Incorporate personal and environmental factors into each residents’ care plan and daily routine - Use a biographical approach to capture the residents’ motives for activities and participation as a basis for the personal goal planning - Address environmental factors:   - Optimise the provision of adaptive technologies, mobility aids, and other devices and physio/occupational therapy   - Involve family members or volunteers in planning activities | Organisational level   - Review the need for action at the organisational level using a checklist - Integrate focus on activities and participation in the individual care plans - Dissemination of the PECAN concept to the (visiting) staff and the public - Focus on interprofessional collaborations between care, social care, and physio/occupational therapists - Review and work on environmental factors indoors and outdoors |
