# Supplementary material for: Process evaluation of an individually tailored complex intervention to improve activities and participation of older nursing home residents with joint contractures (JointConEval): a mixed-methods study
Source: Trials. 2024 Dec 18;25:831. doi: 10.1186/s13063-024-08652-2 (PMC11654093; doi:10.1186/s13063-024-08652-2)
Supplement: Supplementary file 2 — Additional file 2. Sample characteristics of interview and focus group participants. [file 13063_2024_8652_MOESM2_ESM.docx]

Additional file 2. Sample characteristics of interview and focus group participants

| **Characteristics** | **Numbers (%)*** |
| --- | --- |
| **Facilitators** (n=30) |  |
| Age groups, years |  |
| 20-29 | 1 (3) |
| 30-39 | 18 (60) |
| 40-49 | 4 (13) |
| 50-59 | 7 (23) |
| Female gender | 22 (73) |
| Professional background |  |
| Skilled nurse | 6 (20) |
| Skilled geriatric nurse | 19 (63) |
| Occupational therapist | 4 (13) |
| Management | 1 (3) |
| Leading position | 17 (57) |
| Professional experience in geriatric care, *years* |  |
| 1-5 | 2 (7) |
| 6-10 | 6 (20) |
| ≥11 | 22 (73) |
| **Nursing and social care staff** (n=78) |  |
| Mean age, *years (range)* | 41.8 (18-68) |
| Female gender | 67 (86) |
| Professional background |  |
| Skilled nurse | 8 (10) |
| Skilled geriatric nurse | 44 (56) |
| Nursing assistant | 6 (8) |
| Geriatric nursing student | 4 (5) |
| Social pedagogue | 1 (1) |
| Social care assistant | 13 (17) |
| Occupational therapist | 1 (1) |
| Physiotherapist | 1 (1) |
| Leading position | 20 (26) |
| Professional experience in (geriatric) care, *years* |  |
| ≤ 1 | 3 (4) |
| 1-5 | 13 (17) |
| 6-10 | 17 (22) |
| ≥11 | 42 (54) |
| **Nursing home residents** (n=35) |  |
| Mean age, *years (range)* | 83.7 (71-97) |
| Female gender | 30 (86) |
| **Relatives/guardians** (n=10) |  |
| Mean age, *years (range)* | 62.5 (50-74) |
| Female gender | 7 (70) |
| Relationship to resident |  |
| Spouse | 1 (10) |
| Child | 8 (80) |
| Guardian | 1 (10) |
| Occupation |  |
| Employed | 6 (60) |
| Retired | 4 (40) |
| *Values are numbers (percentages) unless stated otherwise |  |
