# Supplementary material for: Process evaluation of an individually tailored complex intervention to improve activities and participation of older nursing home residents with joint contractures (JointConEval): a mixed-methods study
Source: Trials. 2024 Dec 18;25:831. doi: 10.1186/s13063-024-08652-2 (PMC11654093; doi:10.1186/s13063-024-08652-2)
Supplement: Supplementary file 3 — Additional file 3. Sample characteristics of questionnaire participants: Nursing and social care staff. [file 13063_2024_8652_MOESM3_ESM.docx]

**Additional file 3.** Sample characteristics of questionnaire participants: Nursing and social care staff

|  | **Baseline** | | **12-month follow up** | |
| --- | --- | --- | --- | --- |
| **Characteristics** | Intervention group, n=187 (%)* | Control group  n=172 (%)* | Intervention group n=194 (%)* | Control group  n=200 (%)* |
| Professional background | *n=181* | *n=164* | *n=187* | *n=190* |
| Skilled (geriatric) nurse | 80 (44.2) | 85 (51.8) | 97 (51.9) | 100 (52.6) |
| Nursing assistant | 53 (29.3) | 40 (24.4) | 49 (26.2) | 53 (27.9) |
| (Geriatric) Nursing student | 13 (7.2) | 9 (5.5) | 6 (3.2) | 6 (3.2) |
| Skilled nurse, academic degree | 4 (2.2) | 5 (3.0) | 3 (1.6) | 5 (2.6) |
| Social care assistant | 31 (17.1) | 25 (15.2) | 32 (17.1) | 26 (13.7) |
| Average weekly working time, *mean* *hours, (range)* | *n=176*  33.9 (7-40) | *n=166*  35.6 (7-40) | *n=185*  32.9 (7-40) | *n=196*  35.4 (8-40) |
| Working years/facility | *n=187* | *n=171* | *n=191* | *n=198* |
| ≤ 1 | 26 (13.9) | 21 (12.3) | 14 (7.3) | 27 (13.6) |
| 1-5 | 80 (42.8) | 57 (33.3) | 75 (39.3) | 75 (37.9) |
| 6-10 | 40 (21.4) | 43 (25.1) | 47 (24.6) | 36 (18.2) |
| ≥11 | 41 (21.9) | 50 (29.2) | 55 (28.8) | 60 (30.3) |
| *Values are numbers (percentages) unless stated otherwise | | | | |
