# Supplementary material for: Process evaluation of an individually tailored complex intervention to improve activities and participation of older nursing home residents with joint contractures (JointConEval): a mixed-methods study
Source: Trials. 2024 Dec 18;25:831. doi: 10.1186/s13063-024-08652-2 (PMC11654093; doi:10.1186/s13063-024-08652-2)
Supplement: Supplementary file 4 — Additional file 4. Detailed overview of the delivery in the clusters. [file 13063_2024_8652_MOESM4_ESM.docx]

Additional file 4. Detailed overview of the delivery in the clusters

|  | **Cluster 1** | **Cluster 2** | **Cluster 3** | **Cluster 4** | **Cluster 5** | **Cluster 6** | **Cluster 7** | **Cluster 8** | **Cluster 9** | **Cluster 10** | **Cluster 11** | **Cluster 12** | **Cluster 13** | **Cluster 14** | **Cluster 15** | **Cluster 16** | **Cluster 17** | **Cluster 18** |  |
| --- | --- | --- | --- | --- | --- | --- | --- | --- | --- | --- | --- | --- | --- | --- | --- | --- | --- | --- | --- |
| **1) Kick-off meeting** |  | | | | | | | | | | | | | | | | | |  |
| Number of participants, n=28 | 2 | 1 | 1 | 1 | 1 | 2 | 1 | 1 | 1 | 1 | 1 | 4 | 2 | 1 | 1 | 2 | 3 | 2 |  |
| *Participation of at least one manager* | ✓ | ✓ | ✓ | ✓ | ✓ | ✓ | ✓ | ✓ | ✓ | ✓ | ✓ | ✓ | ✓ | ✓ | ✓ | ✓ | ✓ | ✓ |  |
| *Number of nominated facilitators, n=39* | 3 | 2 | 1 | 2 | 3 | 2 | 2 | 2 | 2 | 2 | 2 | 2 | 2 | 2 | 2 | 2 | 2 | 4 |  |
| *Managers signed declaration* | ✓ | ✓ | ✓ | ✓ | ✓ | ✓ | ✓ | ✓ | ✓ | ✓ | ✓ | ✓ | ✓ | ✓ | ✓ | ✓ | ✓ | ✓ |  |
| *Meeting conducted according to protocol* | ✓ | ✓ | ✓ | ✓ | ✓ | ✓ | Ø | Ø | Ø | ✓ | ✓ | ✓ | ✓ | ✓ | ✓ | ✓ | ✓ | ✓ |  |
| *Satisfaction with delivery rated by researchers* | ↑ | ↑ | ↓ | ↑ | ↑ | ↑ | → | ↓ | ↓ | → | ↑ | ↑ | → | ↑ | ↑ | ↑ | → | ↑ |  |
| **Domain total in %** | **100** | **100** | **67** | **100** | **100** | **100** | **67** | **56** | **56** | **89** | **100** | **100** | **89** | **100** | **100** | **100** | **89** | **100** |  |
| **Score (weight=0.5)** | **50** | **50** | **33** | **50** | **50** | **50** | **33** | **28** | **28** | **44** | **50** | **50** | **44** | **50** | **50** | **50** | **44** | **50** |  |
| **2) Facilitators’ workshop** |  | | | | | | | | | | | | | | | | | |  |
| Number of participants, n=45 | 4 | 2 | 1 | 3 | 5 | 1 | 2 | 2 | 2 | 4 | 2 | 2 | 2 | 2 | 2 | 4 | 2 | 3 |  |
| *Number of trained facilitators in relation to total nominated facilitators, n=37* | 3/3 | 2/2 | 1/1 | 2/2 | 3/3 | 1/2 | 2/2 | 2/2 | 2/2 | 2/2 | 2/2 | 2/2 | 2/2 | 2/2 | 2/2 | 2/2 | 2/2 | 3/4 |  |
| Achievement of learning objectives rated by researchers: |  | | | | | | | | | | | | | | | | | |  |
| *Recognise the relevance of the topic* | ↑ | ↑ | ↑ | ↑ | ↑ | ↑ | ↑ | ↑ | ↑ | ↓ | ↓ | ↓ | ↓ | ↑ | ↑ | ↑ | ↑ | ↑ |  |
| *Be aware of the consequences of joint contractures* | ↑ | ↑ | ↑ | ↑ | ↑ | ↑ | ↑ | ↑ | ↑ | ↑ | ↑ | ↑ | ↑ | ↑ | ↑ | ↑ | ↑ | ↑ |  |
| *Know strategies for the involvement of relevant healthcare professions* | → | → | → | → | → | → | → | → | → | → | → | → | → | → | → | → | → | → |  |
| *Agenda and content according to protocol* | ✓ | ✓ | ✓ | ✓ | ✓ | ✓ | ✓ | ✓ | ✓ | ✓ | ✓ | ✓ | ✓ | ✓ | ✓ | ✓ | ✓ | ✓ |  |
| *Satisfaction with delivery as rated by researchers* | ↑ | ↑ | ↑ | ↑ | ↑ | ↑ | ↑ | ↑ | ↑ | → | → | → | → | ↑ | ↑ | ↑ | ↑ | ↑ |  |
| **Domain total in %** | **92** | **92** | **92** | **92** | **92** | **83** | **92** | **92** | **92** | **67** | **67** | **67** | **67** | **92** | **92** | **92** | **92** | **83** |  |
| **Score (weight=1)** | **92** | **92** | **92** | **92** | **92** | **83** | **92** | **92** | **92** | **67** | **67** | **67** | **67** | **92** | **92** | **92** | **92** | **83** |  |
| **3) Peer mentor approach** |  | | | | | | | | | | | | | | | | | |  |
| Peer mentor visit |  | | | | | | | | | | | | | | | | | |  |
| Number of facilitators participating in relation to total facilitators, n=33 | 2/3 | 2/2 | 1/1 | 1/2 | 3/3 | 2/2 | 2/2 | 1/2 | 2/2 | 2/2 | 2/2 | 2/2 | 2/2 | 2/2 | 1/2 | 1/2 | 2/2 | 3/4 |  |
| Number of managers participating, n=20 | 1 | 1 | 0 | 1 | 2 | 1 | 1 | 1 | 1 | 1 | 1 | 1 | 2 | 1 | 1 | 1 | 2 | 1 |  |
| *Adequate preparation of the cluster at individual level* | ↑ | ↑ | ↑ | ↑ | ↑ | ↑ | ↑ | ↓ | ↑ | ↑ | ↑ | ↓ | ↑ | ↓ | ↑ | ↑ | ↑ | ↑ |  |
| *Adequate preparation of the cluster at organisational level* | ↑ | ↑ | ↓ | ↓ | ↑ | ↑ | ↑ | ↓ | ↑ | ↓ | ↑ | ↓ | ↑ | ↑ | ↑ | ↑ | ↑ | ↑ |  |
| *Agenda and content according to protocol* | ✓ | ✓ | ✓ | ✓ | ✓ | ✓ | ✓ | ✓ | ✓ | ✓ | ✓ | ✓ | ✓ | ✓ | ✓ | ✓ | ✓ | ✓ |  |
| *Satisfaction with delivery as rated by researchers* | ↑ | ↑ | ↓ | ↑ | ↑ | ↑ | ↑ | ↑ | ↑ | ↑ | ↑ | ↓ | ↑ | ↑ | ↑ | ↓ | ↑ | ↑ |  |
| Number of additional consultations on-site, n=8 | 1 | 1 | 2 | 1 | 0 | 0 | 0 | 0 | 1 | 0 | 0 | 0 | 2 | 0 | 0 | 0 | 0 | 0 |  |
| **Domain total in %** | **89** | **100** | **56** | **78** | **100** | **100** | **100** | **67** | **100** | **89** | **100** | **56** | **100** | **89** | **89** | **67** | **100** | **89** |  |
| **Score (weight=1.5)** | **133** | **150** | **83** | **117** | **150** | **150** | **150** | **100** | **150** | **133** | **150** | **83** | **150** | **133** | **133** | **100** | **150** | **133** |  |
| Peer counselling via telephone^a^ |  | | | | | | | | | | | | | | | | | |  |
| *Number of telephone consultations with facilitators^b^, n=160* | 5/11 | 7/11 | 12/11 | 14/11 | 6/11 | 16/11 | 11/11 | 7/11 | 9/11 | 9*/11 | 9/11 | 0/11 | 11/11 | 3*/11 | 10*/11 | 10*/11 | 10*/11 | 11*/11 |  |
| *Number of facilitators counselled in relation to total facilitators, n=30* | 2/3 | 2/2 | 1/1 | 2/2 | 2/3 | 2/2 | 2/2 | 2/2 | 2/2 | 2/2 | 2/2 | 0 | 2/2 | 1/2 | 1/2 | 1/2 | 1/2 | 3/4 |  |
| Duration of counselling session of facilitators in minutes, mean (range) | 19 (10-30) | 41  (25-60) | 24  (10-40) | 23  (10-45) | 19  (10-30) | 31  (3-60) | 32  (10-45) | 42  (20-60) | 43  (30-70) | 46  (30-60) | 38  (30-60) | 0 | 32  (20-45) | 33  (30-40) | 35  (20-60) | 28  (20-40) | 38  (20-60) | 35  (15-60) |  |
| Number of residents discussed in relation to total participating residents, n=120 | 3/11 | 8/13 | 8/21 | 8/16 | 2/13 | 18/20 | 10/15 | 8/19 | 7/17 | 12/16 | 5/15 | 0/22 | 8/17 | 2/17 | 5/15 | 5/17 | 5/19 | 6/18 |  |
| *Number of telephone consultations with managers, n=21* | 3 | 0 | 2 | 4 | 4 | 1 | 1 | 1 | 1 | n.a.* | 2 | 0 | 2/2 | n.a* | n.a* | n.a* | n.a* | n.a* |  |
| Counselling duration of managers in minutes, mean (range) | 28  (15-40) | 0 | 30  (25-35) | 29  (15-45) | 19  (15-25) | 30 | 35 | 30 | 15 | n.a.* | 15  (15) | 0 | 40  (20-60) | n.a* | n.a* | n.a* | n.a* | n.a* |  |
| **Domain total in %** | **50** | **50** | **100** | **100** | **67** | **100** | **100** | **83** | **83** | **83** | **83** | **0** | **100** | **50** | **67** | **67** | **67** | **83** |  |
| **Score (weight=2)** | **100** | **100** | **200** | **200** | **133** | **200** | **200** | **167** | **167** | **167** | **167** | **0** | **200** | **100** | **133** | **133** | **133** | **167** |  |
| **4) Information session** |  | | | | | | | | | | | | | | | | | |  |
| Invitation of: |  | | | | | | | | | | | | | | | | | |  |
| *Nursing staff* | ✓ | ✓ | ✓ | Ø | ✓ | ✓ | ✓ | ✓ | ✓ | ✓ | ✓ | ✓ | ✓ | ✓ | ✓ | ✓ | ✓ | ✓ |  |
| *Social care staff* | ✓ | ✓ | ✓ | Ø | ✓ | ✓ | Ø | Ø | Ø | ✓ | ✓ | ✓ | ✓ | ✓ | ✓ | ✓ | ✓ | ✓ |  |
| *Type of invitation:* |  | | | | | | | | | | | | | | | | | |  |
| *Personal (face to face)* | ✓ | ✓ | Ø | ✓ | ✓ | Ø | ✓ | Ø | Ø | ✓ | Ø | ✓ | ✓ | ✓ | ✓ | ✓ | ✓ | ✓ |  |
| *Letter or mail* | Ø | Ø | Ø | Ø | ✓ | Ø | Ø | Ø | Ø | Ø | ✓ | ✓ | ✓ | Ø | Ø | Ø | ✓ | ✓ |  |
| *Flyer, notice board, homepage* | ✓ | ✓ | ✓ | Ø | Ø | ✓ | Ø | ✓ | ✓ | Ø | Ø | Ø | Ø | ✓ | ✓ | ✓ | ✓ | ✓ |  |
| Number of participants: |  | | | | | | | | | | | | | | | | | |  |
| Nursing staff in relation to total nursing staff, n=186 | 14/37 | 4/29 | 12/38 | 9/24 | 8/36 | 15/38 | 7/30 | 8/34 | 7/63 | 18/80 | 3/54 | 15/54 | 9/15 | 10/42 | 10/36 | 14/48 | 4/54 | 19/51 |  |
| Social care staff in relation to total social care staff, n=53 | 3/7 | 1/4 | 3/7 | 2/4 | 1/6 | 2/6 | 0/3 | 4/6 | 1/10 | 11/12 | 3/9 | 0/9 | 3/7 | 5/6 | 2/6 | 4/7 | 1/6 | 7/8 |  |
| Therapists, n=19 | 0 | 0 | 0 | 1 | 5 | 1 | 2 | 2 | 1 | 0 | 0 | 1 | 0 | 0 | 4 | 2 | 0 | 0 |  |
| Residents, n=21 | 0 | 0 | 0 | 0 | 0 | 0 | 2 | 0 | 9 | 0 | 0 | 0 | 0 | 0 | 0 | 10 | 0 | 0 |  |
| Relatives, n=10 | 5 | 0 | 0 | 0 | 0 | 0 | 3 | 0 | 0 | 0 | 0 | 0 | 0 | 1 | 0 | 0 | 1 | 0 |  |
| Others, n=57 | 5 | 0 | 1 | 3 | 2 | 3 | 3 | 2 | 5 | 3 | 7 | 1 | 2 | 1 | 7 | 3 | 5 | 4 |  |
| *Total, n=346* | 27 | 5 | 16 | 15 | 16 | 21 | 17 | 16 | 23 | 32 | 13 | 17 | 14 | 17 | 23 | 33 | 11 | 30 |  |
| Achievement of learning objectives rated by researchers: |  | | | | | | | | | | | | | | | | | |  |
| *Informed about PECAN and its objectives* | ↑ | ↑ | → | → | ↑ | ↑ | → | ↑ | → | ↑ | ↑ | ↑ | ↑ | → | ↑ | → | → | ↓ |  |
| *Awareness of own roles in the implementation of PECAN* | → | → | → | → | → | ↑ | → | ↑ | → | → | ↓ | ↓ | ↓ | → | → | → | ↓ | ↓ |  |
| *Session conducted according to the protocol* | ✓ | ✓ | ✓ | ✓ | ✓ | ✓ | ✓ | ✓ | ✓ | ✓ | ✓ | ✓ | ✓ | ✓ | ✓ | ✓ | ✓ | ✓ |  |
| *Satisfaction with delivery rated by researchers* | ↑ | ↑ | ↑ | ↑ | ↑ | ↑ | ↑ | ↑ | ↑ | ↑ | → | → | → | ↑ | ↑ | ↑ | → | → |  |
| **Domain total in %** | **92** | **92** | **75** | **58** | **92** | **92** | **67** | **83** | **67** | **83** | **67** | **75** | **75** | **83** | **92** | **83** | **67** | **58** |  |
| **Score (weight=1)** | **92** | **92** | **75** | **58** | **92** | **92** | **67** | **83** | **67** | **83** | **67** | **75** | **75** | **83** | **92** | **83** | **67** | **58** |  |
| **5) In-house information event** |  | | | | | | | | | | | | | | | | | |  |
| *Invitation of:* |  | | | | | | | | | | | | | | | | | |  |
| *Residents* | Ø | Ø | Ø | Ø | Ø | Ø | ✓ | ✓ | ✓ | Ø | Ø | Ø | Ø | Ø | Ø | ✓ | Ø | Ø |  |
| *Relatives* | ✓ | Ø | Ø | Ø | Ø | Ø | ✓ | Ø | ✓ | Ø | Ø | Ø | Ø | ✓ | Ø | ✓ | ✓ | ✓ |  |
| *Therapists* | ✓ | Ø | Ø | Ø | ✓ | Ø | ✓ | Ø | Ø | Ø | Ø | ✓ | Ø | Ø | Ø | ✓ | Ø | ✓ |  |
| *Type of invitation:* |  | | | | | | | | | | | | | | | | | |  |
| *Personal (face to face)* | ✓ | ✓ | Ø | Ø | ✓ | Ø | ✓ | Ø | Ø | ✓ | Ø | ✓ | ✓ | ✓ | ✓ | ✓ | ✓ | ✓ |  |
| *Letter or mail* | Ø | Ø | Ø | Ø | ✓ | Ø | Ø | Ø | Ø | Ø | ✓ | ✓ | ✓ | Ø | Ø | Ø | ✓ | ✓ |  |
| *Flyer, notice board, homepage* | ✓ | ✓ | ✓ | Ø | Ø | ✓ | Ø | ✓ | ✓ | Ø | Ø | Ø | Ø | ✓ | ✓ | ✓ | ✓ | ✓ |  |
| Number of participants: |  | | | | | | | | | | | | | | | | | |  |
| Nursing home staff, n=31 | 1 | 0 | 0 | 1 | 3 | 0 | 1 | 1 | 1 | 6 | 1 | 6 | 2 | 2 | 0 | 0 | 3 | 3 |  |
| Residents, n=67 | 2 | 1 | 0 | 2 | 0 | 1 | 1 | 2 | 9 | 5 | 0 | 5 | 11 | 3 | 4 | 6 | 7 | 8 |  |
| Relatives, n=50 | 3 | 0 | 0 | 2 | 0 | 1 | 2 | 1 | 0 | 9 | 4 | 8 | 2 | 4 | 2 | 3 | 1 | 8 |  |
| Visiting health care professionals, n=16 | 2 | 0 | 2 | 0 | 0 | 0 | 0 | 0 | 0 | 4 | 0 | 1 | 3 | 0 | 0 | 2 | 2 | 0 |  |
| Volunteers, n=7 | 0 | 0 | 0 | 0 | 0 | 0 | 0 | 0 | 0 | 1 | 0 | 0 | 1 | 2 | 0 | 0 | 1 | 2 |  |
| *Total, n=171* | 8 | 1 | 2 | 5 | 3 | 2 | 4 | 4 | 10 | 25 | 5 | 20 | 19 | 11 | 6 | 11 | 14 | 21 |  |
| *Information event conducted according to the protocol* | ✓ | ✓ | ✓ | ✓ | ✓ | ✓ | ✓ | ✓ | ✓ | ✓ | ✓ | ✓ | ✓ | ✓ | ✓ | ✓ | ✓ | ✓ |  |
| *Satisfaction with delivery rated by researchers* | ↑ | ↑ | → | → | ↓ | ↑ | ↑ | ↑ | ↑ | ↑ | → | ↓ | ↑ | → | ↑ | ↓ | → | ↑ |  |
| **Domain total in %** | **89** | **67** | **44** | **33** | **56** | **56** | **89** | **67** | **78** | **56** | **44** | **56** | **67** | **67** | **67** | **78** | **67** | **89** |  |
| **Score (weight=0.5)** | **44** | **33** | **22** | **17** | **28** | **28** | **44** | **33** | **39** | **28** | **22** | **28** | **33** | **33** | **33** | **39** | **33** | **44** |  |
| **6) Facilitators’ exchange and training session** |  | | | | | | | | | | | | | | | | | |  |
| Number of participants, n=34 | 2 | 1 | 1 | 2 | 5 | 1 | 2 | 2 | 2 | 0 | 2 | 0 | 1 | 2 | 2 | 4 | 2 | 3 |  |
| Number of facilitators participating in relation to total facilitators, n=30 | 2/3 | 1/2 | 1/1 | 2/2 | 3/3 | 1/2 | 2/2 | 2/2 | 2/2 | 0/2 | 2/2 | 0/2 | 1/2 | 2/2 | 2/2 | 2/2 | 2/2 | 3/4 |  |
| Achievement of learning objectives rated by researchers: |  | | | | | | | | | | | | | | | | | |  |
| *Acquired skills in counselling colleagues* | ↑ | ↑ | ↑ | ↑ | ↑ | ↑ | ↑ | ↑ | ↑ | n.a | ↑ | n.a | ↑ | → | → | → | → | → |  |
| *Be able to apply methods of collegial counselling* | ↑ | ↑ | ↑ | ↑ | ↑ | ↑ | ↑ | ↑ | ↑ | n.a | ↑ | n.a | ↑ | ↑ | ↑ | ↑ | ↑ | ↑ |  |
| *Exchange experience and gain new impulses for practice* | ↑ | ↑ | ↑ | ↑ | ↑ | ↑ | ↑ | ↑ | ↑ | n.a | ↓ | n.a | ↑ | ↑ | ↑ | ↑ | ↑ | ↑ |  |
| *Agenda and content according to protocol* | ✓ | ✓ | ✓ | ✓ | ✓ | ✓ | ✓ | ✓ | ✓ | Ø | ✓ | Ø | ✓ | ✓ | ✓ | ✓ | ✓ | ✓ |  |
| *Satisfaction with delivery rated by researchers* | ↑ | ↑ | ↑ | ↑ | ↑ | ↑ | ↑ | ↑ | ↑ | n.a | → | n.a | → | ↑ | ↑ | ↑ | ↑ | ↑ |  |
| **Domain total in %** | **92** | **92** | **100** | **100** | **100** | **92** | **100** | **100** | **100** | **0** | **75** | **0** | **83** | **92** | **92** | **92** | **92** | **83** |  |
| **Score (weight=1)** | **92** | **92** | **100** | **100** | **100** | **92** | **100** | **100** | **100** | **0** | **75** | **0** | **83** | **92** | **92** | **92** | **92** | **83** |  |
| **Total weight score all domains** | **603** | **608** | **606** | **633** | **644** | **694** | **686** | **603** | **642** | **522** | **597** | **303** | **653** | **583** | **625** | **589** | **611** | **619** |  |
| **Total score in percent^c^** | **80** | **81** | **81** | **84** | **86** | **93** | **91** | **80** | **86** | **70** | **80** | **40** | **87** | **78** | **83** | **79** | **81** | **83** |  |

Legend: ✓ = done; Ø = none; ↑= approval or satisfaction, → = partly or neither, ↓ = no / little agreement or satisfaction; n.a. = not applicable; * = Consultation at the organisational level in combination with consultation at the individual level; Items in italics have been included in the scoring

^a^ A total of eleven telephone consultations were planned for the delivery. However, the consultations were planned individually per cluster, which is why the number differs.

^b^ At least 11 consultations per cluster were planned, but additional consultations were possible.

^c^ A delivery score was calculated for each implementation component and is presented here as percentage of the maximum total score. mean=80; min=40; max=93
