# Supplementary material for: Process evaluation of an individually tailored complex intervention to improve activities and participation of older nursing home residents with joint contractures (JointConEval): a mixed-methods study
Source: Trials. 2024 Dec 18;25:831. doi: 10.1186/s13063-024-08652-2 (PMC11654093; doi:10.1186/s13063-024-08652-2)
Supplement: Supplementary file 5 — Additional file 5. Delivery scoring system. [file 13063_2024_8652_MOESM5_ESM.docx]

Additional file 5. Delivery scoring system

| **Implementation steps** | | | **Scoring system** | | | | |
| --- | --- | --- | --- | --- | --- | --- | --- |
| *1) Kick-off meeting* | | | *Domain total: 9 points (=100%)*  *Maximum weighted score (weight=0.5): 50* | | | | |
| Participation of at least one manager | | | 1 point: at least one manager has participated | | | | |
| Facilitators were nominated | | | 2 points: 2 facilitators were nominated, 1 point: 1 facilitator was nominated | | | | |
| Declaration was signed by managers | | | 2 points: manager signed the declaration during the kick-off | | | | |
| Meeting conducted according to protocol | | | 2 points: conducted on-site, 1 point: conducted by telephone | | | | |
| Satisfaction with delivery rated by researchers | | | 2 points: full satisfaction  1 point: partial satisfaction | | | | |
| 2) Facilitators’ workshop | | | *Domain total: 12 points (=100%)*  *Maximum weighted score (weight=1): 100* | | | | |
| Number of participating facilitators | | | 2 points: all nominated facilitators participated,  1 point: at least 1 facilitator participated | | | | |
| *Achievement of learning objectives rated by researchers:* | | |  | | | | |
| Recognise the relevance of the topic | | | 2 points: fully achieved, 1 point: mostly achieved | | | | |
| Be aware of the consequences of contractures | | | 2 points: fully achieved, 1 point: mostly achieved | | | | |
| Know strategies for the involvement of relevant healthcare professions | | | 2 points: fully achieved, 1 point: mostly achieved | | | | |
| Agenda and content according to protocol | | | 2 points: conducted according to protocol | | | | |
| Satisfaction with delivery rated by researchers | | | 2 points: full satisfaction, 1 point: partial satisfaction | | | | |
| *3) Peer mentor approach* | | |  | | | | |
| *Peer mentor visit* | | | *Domain total: 9 points (=100%)*  *Maximum weighted score (weight=1.5): 150* | | | | |
| Number of participating facilitators | | | 2 points: all facilitators participated, 1 point: at least one facilitator participated | | | | |
| Participation of at least one manager | | | 1 point: at least one manager participated | | | | |
| Adequate preparation of the cluster for the peer mentor visit | | | 2 points: individual level and organisational level were prepared, 1 point: only one level was prepared | | | | |
| Agenda and content according to protocol | | | 2 points: conducted according to protocol | | | | |
| Satisfaction with delivery rated by researchers | | | 2 points: full satisfaction, 1 point: partial satisfaction | | | | |
| *Peer counselling via telephone* | | | *Domain total: 6 points (=100%)*  *Maximum weighted score (weight=2): 200* | | | | |
| Number of telephone consultations with facilitators carried out in relation to total planned telephone consultations | | | 2 points: all planned (11) consultations have taken place,  1 point: at least 6 of the planned consultations have taken place | | | | |
| Number of facilitators counselled in relation to total facilitators | | | 2 points: all facilitators were consulted by telephone, 1 point: at least one facilitator was consulted by telephone | | | | |
| Number of telephone consultations with managers | | | 2 points: at least one consultation with a manager has taken place | | | | |
| *4) Information session* | | | *Domain total: 12 points (=100%)*  *Maximum weighted score (weight=1): 100* | | | | |
| Invitation of: | | |  | | | | |
| Nursing staff | | | 1 point: invitation issued | | | | |
| Social care staff | | | 1 point: invitation issued | | | | |
| Type of invitation | | | 2 points: use of different information channels for invitations, 1 point: use of only one information channel | | | | |
|  | | |  | | | | |
| *Achievement of learning objectives rated by researchers:* | | |  | | | | |
| Informed about PECAN and its objectives | | | 2 points: fully achieved, 1 point: mostly achieved | | | | |
| Awareness of own roles in the implementation of PECAN | | | 2 points: fully achieved, 1 point: mostly achieved | | | | |
| Session conducted according to protocol | | | 2 points: conduct in accordance to protocol | | | | |
| Satisfaction with delivery rated by researchers | | | 2 points: full satisfaction, 1 point: partial satisfaction | | | | |
| *5) In-house information event* | | | *Domain total: 9 points (=100%)*  *Maximum weighted score (weight=0.5): 50* | | | | |
| Invitation of: | | |  | | | | |
| Residents | | | 1 point: invitation issued | | | | |
| Relatives | | | 1 point: invitation issued | | | | |
| Therapists/visiting healthcare professionals | | | 1 point: invitation issued | | | | |
| Type of invitation | | | 2 points: use of different information channels for invitations, 1 point: use of only one information channel | | | | |
| Information event conduct according to protocol | | | 2 points: conduct according to protocol | | | | |
| Satisfaction with delivery rated by researchers | | | 2 points: full satisfaction, 1 point: partial satisfaction | | | | |
| *6) Facilitator’s exchange and training session* | | | *Domain total: 12 points (=100%)*  *Maximum weighted score (weight=1): 100* | | | | |
| Participation of at least one facilitator | | | 2 points: all facilitators participated, 1 point: at least one facilitator participated | | | | |
| *Achievement of learning objectives rated by researchers:* | | |  | | | | |
| Acquired skills in counselling colleagues | | | 2 points: fully achieved, 1 point: mostly achieved | | | | |
| Be able to apply methods of collegial counselling | | | 2 points: fully achieved, 1 point: mostly achieved | | | | |
| Exchange experience and gain new impulses for practice | | | 2 points: fully achieved, 1 point: mostly achieved | | | | |
| Agenda and content according to protocol | | | 2 points: conduct in accordance with the protocol | | | | |
| Satisfaction with delivery rated by researchers | | | 2 points: full satisfaction, 1 point: partial satisfaction | | | | |
| Definition of colour gradient: |  | *Good delivery* | |  | *Fair delivery* |  | *Poor delivery* |
| 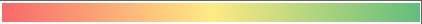 | | | | | | | |
| Maximum domain points = 69 points  Maximum total weight score = 750 | | | | | | | |
