# Supplementary material for: Process evaluation of an individually tailored complex intervention to improve activities and participation of older nursing home residents with joint contractures (JointConEval): a mixed-methods study
Source: Trials. 2024 Dec 18;25:831. doi: 10.1186/s13063-024-08652-2 (PMC11654093; doi:10.1186/s13063-024-08652-2)
Supplement: Supplementary file 6 — Additional file 6. Characteristics of facilitators’ workshop participants. [file 13063_2024_8652_MOESM6_ESM.docx]

Additional file 6. Characteristics of facilitators’ workshop participants

| **Characteristics** | **Numbers (%)***  n=45 |
| --- | --- |
| **Professional background^a^** |  |
| Skilled geriatric nurse | 28 (62.2) |
| Skilled nurse | 9 (20) |
| Other qualifications^b^ | 12 (26.7) |
| **Job position in the nursing home** |  |
| Nurse with management tasks | 13 (28.9) |
| Skilled/skilled geriatric nurse | 10 (22.2) |
| Head nurse | 9 (20) |
| Nurse with mentorship tasks | 4 (8.9) |
| Other positions^c^ | 9 (20) |
| **Professional experience in (geriatric) care**, *years* |  |
| 1 – 5  6 – 10  ≥ 11 | 7 (15.6)  6 (13.3)  32 (71.1) |
| **Average weekly hours of work (n=44)**  Median [min, max]  Mean [SD] | 39 [21; 40]  36.27 [4.9] |

*Values are numbers (percentages) unless stated otherwise

^a^ multiple answers possible

^b^ occupational therapist (n=5); social care staff (n=2); physiotherapist (n=1); other (n=4)

^c^ occupational therapist (n=3); social care staff (n=2); physiotherapist (n=1); other (n=3)
