# Supplementary material for: Process evaluation of an individually tailored complex intervention to improve activities and participation of older nursing home residents with joint contractures (JointConEval): a mixed-methods study
Source: Trials. 2024 Dec 18;25:831. doi: 10.1186/s13063-024-08652-2 (PMC11654093; doi:10.1186/s13063-024-08652-2)
Supplement: Supplementary file 7 — Additional file 7. Baseline characteristics of nursing homes. [file 13063_2024_8652_MOESM7_ESM.docx]

Additional file 7. Baseline characteristics of nursing homes

| **Characteristics** | **Intervention group**  n=18 (%)* | **Control group**  n=17 (%)* |  |
| --- | --- | --- | --- |
| Ownership |  |  | |
| Non-private | 12 (66.7) | 11 (64.7) | |
| private | 6 (33.3) | 6 (35.3) | |
| Location |  |  | |
| urban | 8 (44.4) | 8 (47.1) | |
| rural | 10 (55.6) | 9 (52.9) | |
| Number of residents, *median (min, max)* | 91.0 (54.0, 137) | 99.0 (44.0, 196) | |
| Number of residents per skilled nurse, *median (min, max)* | 5.38 (4.02, 7.90) | 5.23 (3.88, 11.0) | |
| Local environment^a^ |  |  | |
| Park areas | 18 (100) | 15 (88.2) | |
| Stores (e.g. supermarket, drugstore, pharmacy) | 17 (94.4) | 15 (88.2) | |
| Churches | 16 (88.9) | 11 (64.7) | |
| Café | 14 (77.8) | 15 (88.2) | |
| Other | 13^c^ (72.2) | 7^d^ (41.2) | |
| None of the options | 0 (0) | 1 (5.9) | |
| Environment promoting physical activities inside/outside the facility^b^ | 16 (88.9) | 16 (94.1) | |
| Environment promoting social participation inside/outside the facility^c^ | 18 (100) | 15 (88.2) | |
| Case conferences (regularly or occasionally) | 15 (83.3) | 15 (88.2) | |
| General concepts promoting social participation | 16 (88.9) | 15 (88.2) | |
| Concepts promoting social participation for residents with joint contractures | 8 (44.4) | 5 (29.4) | |
| Standard operating procedures on handling joint contractures | 14 (77.8) | 13 (76.5) | |
| Introduction of nursing practice guideline on mobility | 5 (27.8) | 2 (11.8) | |
| * Values are numbers (percentages) unless stated otherwise  ^a^ Number of clusters for which the characteristic applies  ^b^ Local recreation & culture/sports/food/shopping facilities, meeting centres & contact options, health services, financial services, public transportation  ^c^ Local recreation &culture/sports/food/shopping facilities, contact options | | | |
