# Supplementary material for: Process evaluation of an individually tailored complex intervention to improve activities and participation of older nursing home residents with joint contractures (JointConEval): a mixed-methods study
Source: Trials. 2024 Dec 18;25:831. doi: 10.1186/s13063-024-08652-2 (PMC11654093; doi:10.1186/s13063-024-08652-2)

Additional file 8. Organisational culture of the clusters of the intervention group (IG) and control group (CG) at baseline (t0) and after 12 months (t2)


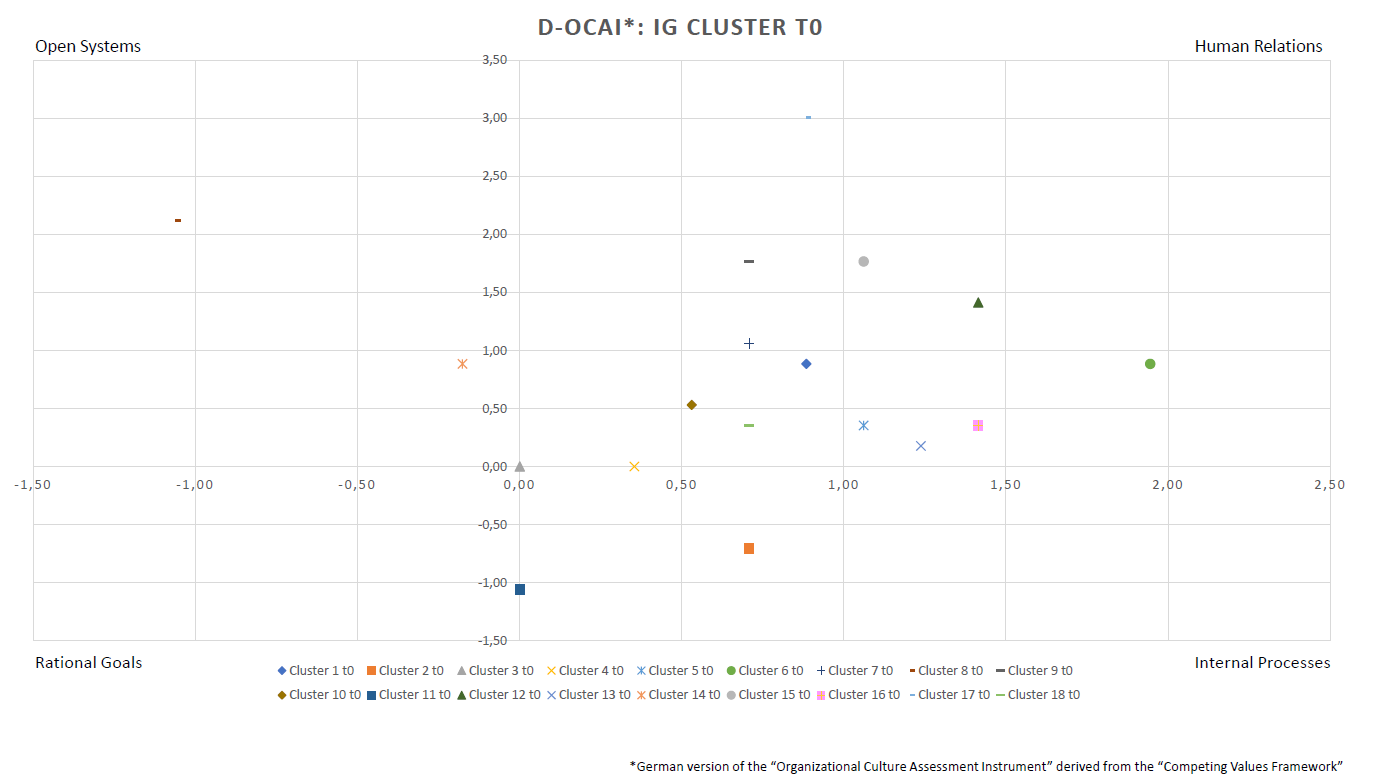


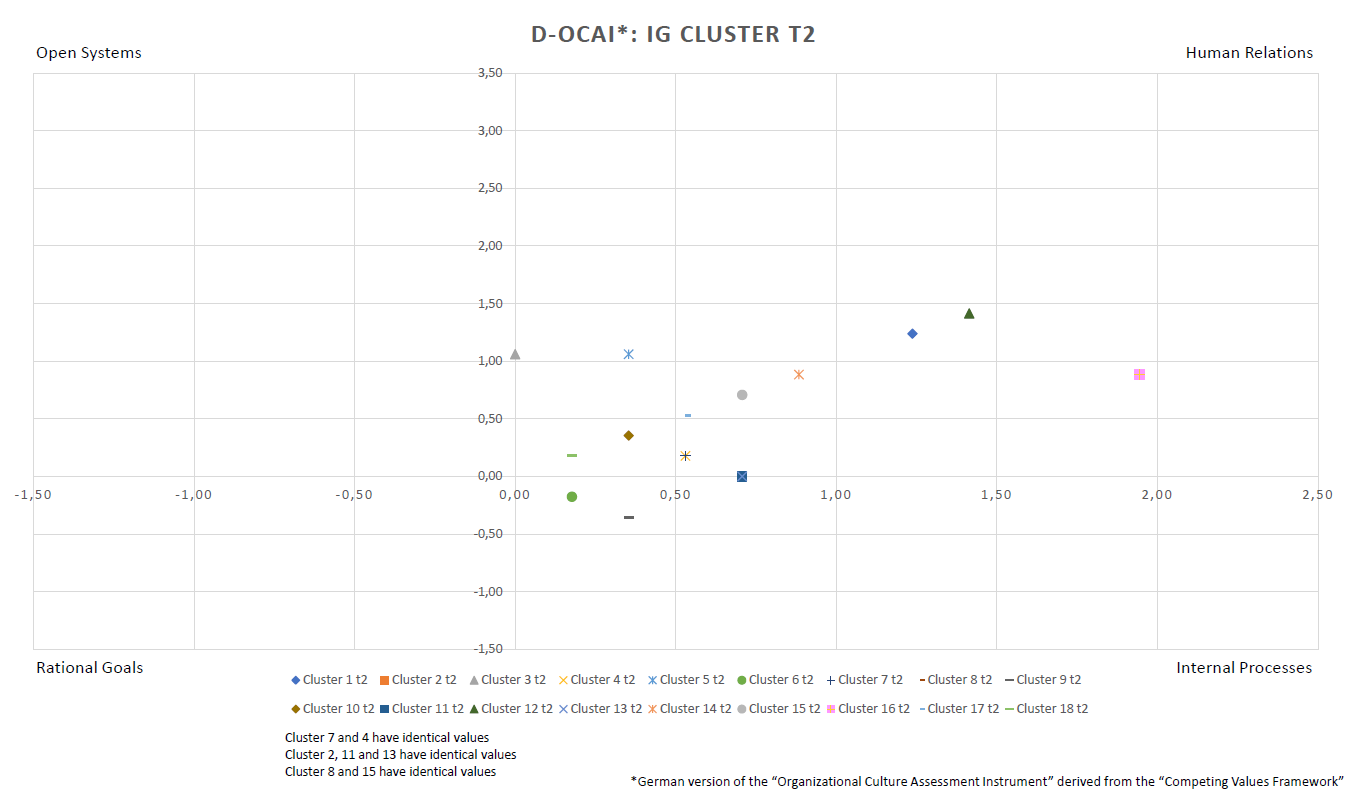


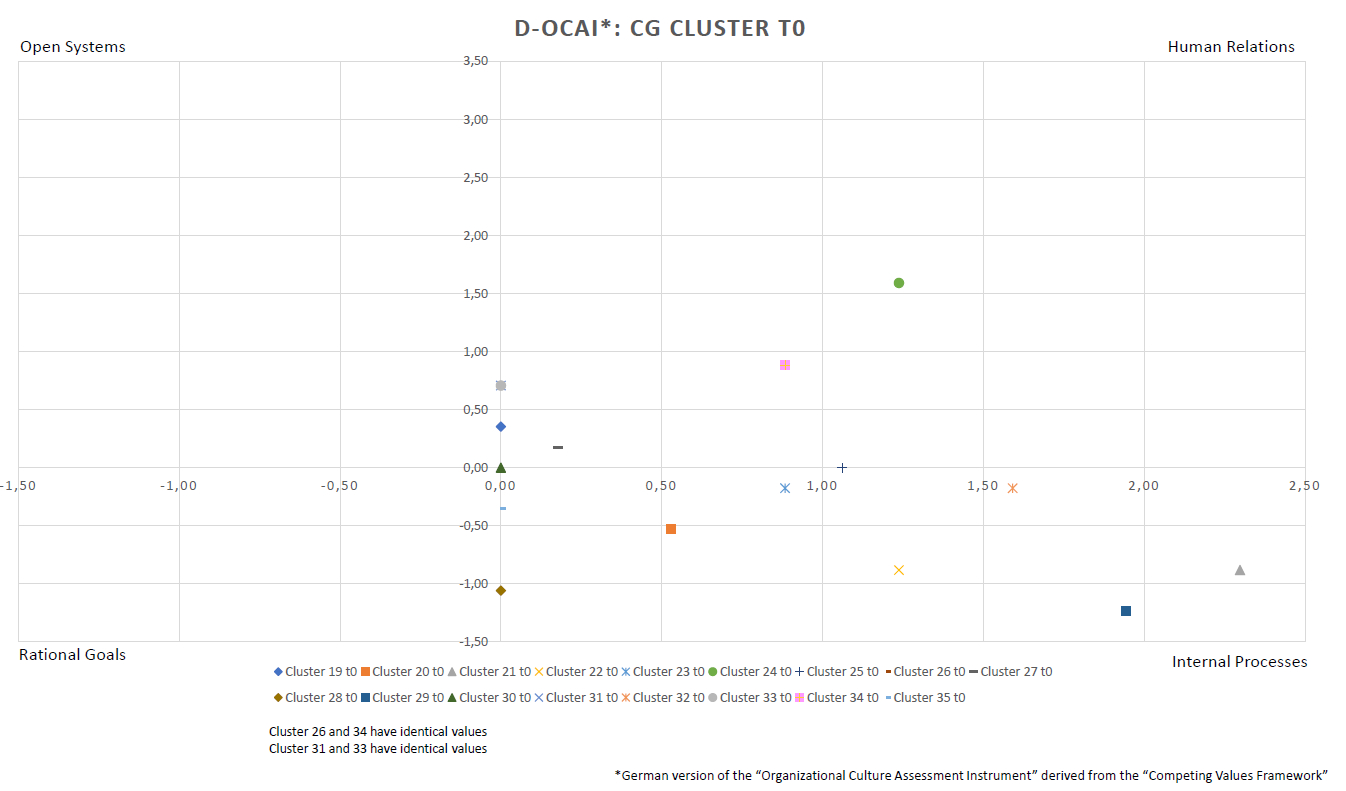


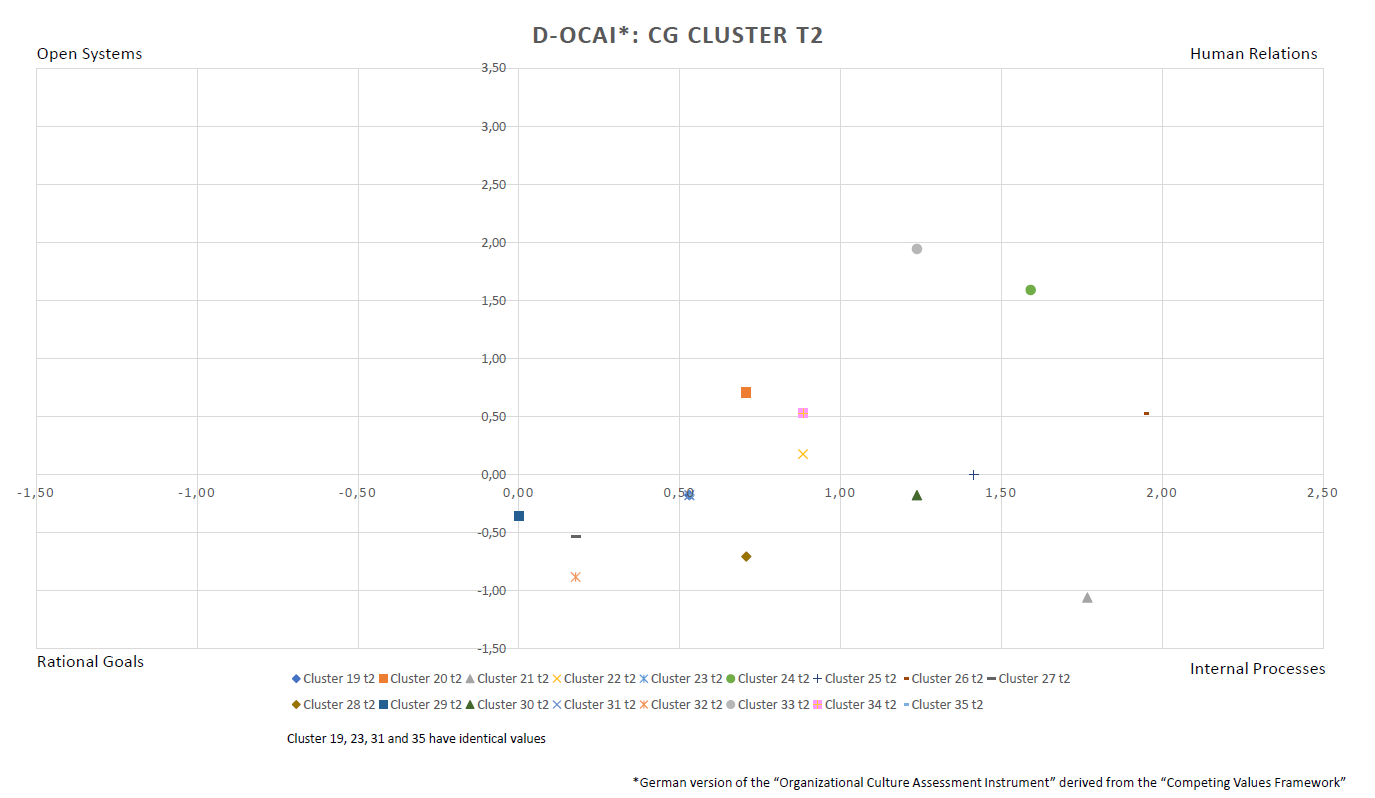

Supplement: Supplementary file 8 — Additional file 8. Organisational culture of the clusters of the intervention group (IG) and control group (CG) at baseline (t0) and after 12 months (t2). [file 13063_2024_8652_MOESM8_ESM.docx]
