# Supplementary material for: Process evaluation of an individually tailored complex intervention to improve activities and participation of older nursing home residents with joint contractures (JointConEval): a mixed-methods study
Source: Trials. 2024 Dec 18;25:831. doi: 10.1186/s13063-024-08652-2 (PMC11654093; doi:10.1186/s13063-024-08652-2)
Supplement: Supplementary file 9 — Additional file 9. Perceived resources and social support in the clusters from the perspective of nursing & social care staff and facilitators to promote activities and participation after 12 months. [file 13063_2024_8652_MOESM9_ESM.docx]

Additional file 9. Perceived resources and social support in the clusters from the perspective of nursing & social care staff and facilitators to promote activities and participation after 12 months

|  | **Agreement by^a^**  **nursing and social care staff** | | **Agreement by**  **facilitators** |
| --- | --- | --- | --- |
|  | **CG^b^, n=200 (%)*** | **IG^c^, n=194 (%)*** | **IG, n=33 (%)*** |
| I have enough time to promote activities and participation. | 57 (28.5) | 75 (38.7) | 15 (45.4) |
| Sufficient human resources are available to promote activities and participation. | 58 (29.0) | 81 (41.8) | 14 (42.4) |
| I receive support in promoting activities and participation by: |  |  |  |
| Supervisors | 122 (61.0) | 128 (66.0) | 30 (90.9) |
| Nursing staff | 136 (68.0) | 151 (77.8) | 21 (63.6) |
| Social care assistants | 124 (62.0) | 137 (70.6) | 28 (84.8) |
| Housekeeping staff | 47 (23.5) | 84 (43.3) | 15 (45.4) |
| Physiotherapists /occupational therapists | 95 (47.5) | 133 (68.6) | 27 (81.8) |
| Physicians | 69 (34.5) | 93 (47.9) | 18 (54.5) |
| Staff of a medical supply store | 70 (35.0) | 87 (44.8) | 22 (66.6) |
| Relatives and/or guardians | 67 (33.5) | 91 (46.9) | 13 (39.4) |

* Values are numbers (percentages) unless stated otherwise

^a^ Includes “I agree” and “I tend to agree”

^b^ CG, control group

^c^ IG, intervention group
